# *Pseudomonas*-associated bacteria play a key role in obtaining nutrition from bamboo for the giant panda (*Ailuropoda melanoleuca*)

**DOI:** 10.1128/spectrum.03819-23

**Published:** 2024-02-02

**Authors:** Ruihong Ning, Caiwu Li, Maohua Xia, Yu Zhang, Yunong Gan, Yan Huang, Tianyou Zhang, Haitao Song, Siyuan Zhang, Wei Guo

**Affiliations:** 1 Sichuan Provincial Engineering Laboratory for Prevention and Control Technology of Veterinary Drug Residue in Animal-origin Food, School of Laboratory Medicine, Chengdu Medical College, Chengdu, China; 2 CAS Key Laboratory of Mountain Ecological Restoration and Bioresource Utilization, Ecological Restoration and Biodiversity Conservation Key Laboratory of Sichuan Province, Chengdu Institute of Biology, Chinese Academy of Sciences, Chengdu, Sichuan, China; 3 Key Laboratory of State Forestry and Grassland Administration (SFGA) on Conservation Biology of Rare Animals in the Giant Panda National Park, The China Conservation and Research Center for the Giant Panda (CCRCGP), Chengdu, China; 4 Beijing Key Laboratory of Captive Wildlife Technology, Beijing Zoo, Beijing, P.R. China; 5 Chimelong Safari Park in Guangdong Province, Guangzhou, China; 6 The Second Affiliated Hospital of Chengdu Medical College, China National Nuclear Corporation 416 Hospital, Chengdu, China; 7 School of Laboratory Medicine, Chengdu Medical College, Chengdu, China; Chengdu University, Chengdu, China

**Keywords:** giant pandas, gut microbiome, lignin degradation, nutrition, adaptive evolution

## Abstract

Gut microbiota plays a vital role in obtaining nutrition from bamboo for giant pandas. However, low cellulase activity has been observed in the panda’s gut. Besides, no specific pathway has been implicated in lignin digestion by gut microbiota of pandas. Therefore, the mechanism by which they obtain nutrients is still controversial. It is necessary to elucidate the precise pathways employed by gut microbiota of pandas to degrade lignin. Here, the metabolic pathways for lignin degradation in pandas were explored by comparing 209 metagenomic sequencing data from wild species with different feeding habits. Lignin degradation central pathways, including beta-ketoadipate and homogentisate pathway, were enriched in the gut of wild bamboo-eating pandas. The gut microbiome of wild bamboo-eating specialists was enriched with genes from pathways implicated in degrading ferulate and *p*-coumarate into acetyl-CoA and succinyl-CoA, which can potentially provide the raw materials for metabolism in pandas. Specifically, *Pseudomonas*, as the most dominant gut bacteria genus, was found to be the main bacteria to provide genes involved in lignin or lignin derivative degradation. Herein, three *Pseudomonas*-associated strains isolated from the feces of wild pandas showed the laccase, lignin peroxidase, and manganese peroxidase activity and extracellular lignin degradation ability *in vitro*. A potential mechanism for pandas to obtain nutrition from bamboo was proposed based on the results. This study provides novel insights into the adaptive evolution of pandas from the perspective of lignin metabolism.

**IMPORTANCE:**

Although giant pandas only feed on bamboo, the mechanism of lignin digestion in pandas is unclear. Here, the metabolic pathways for lignin degradation in wild pandas were explored by comparing gut metagenomic from species with different feeding habits. Results showed that lignin degradation central pathways, including beta-ketoadipate and homogentisate pathway, were enriched in the gut of wild bamboo-eating pandas. Genes from pathways involved in degrading ferulate and *p*-coumarate via beta-ketoadipate pathway were also enriched in bamboo-eating pandas. The final products of the above process, such as acetyl-CoA, can potentially provide the raw materials for metabolism in pandas. Specifically, *Pseudomonas*, as the most dominant gut bacteria genus, mainly provides genes involved in lignin degradation. Herein, *Pseudomonas*-associated strains isolated from the feces of pandas could degrade extracellular lignin. These findings suggest that gut microbiome of pandas is crucial in obtaining nutrition from lignin via *Pseudomonas*, as the main lignin-degrading bacteria.

## INTRODUCTION

Phylogenetic studies have shown that the giant panda (*Ailuropoda melanoleuca*) is an herbivorous mammal belonging to the bear family (1, 2). Giant pandas have undergone a series of evolution to adapt to the special bamboo diet, including ecological (3–5), morphological (6–8), and genetic (9–11) adaptations (4, 12). Notably, giant pandas lack genes for lignin, cellulose, and hemicellulose degrading pathways in their genome (9). As a result, conservation biologists have been curious to know how giant pandas obtain nutrition from bamboo. A complete metabolic pathway for cellulose and hemicellulose degradation has been detected in the intestine of giant pandas (13). Therefore, the gut microbiota is considered the main route through which giant pandas obtain nutrition from bamboo diet (9). However, the gut microbiota of giant pandas has not shown an obvious convergent evolution phenomenon according to their feeding habits, and they still retain a gut microbiota structure like that of carnivores (14–16). Although the abundance of hemicellulose-degrading bacteria in the gut of giant pandas increases after eating bamboo (17), the abundance of genes related to cellulose and hemicellulose degradation in their gut microbiome is significantly lower than that in other herbivores (13, 14, 17). Many previous studies have confirmed that the gut microbiota of giant pandas is distinct from that of red pandas and clusters closer to those of the black bears and carnivores (14–16). However, Huang et al. found a close similarity in gut microbiota structure among bamboo-eating pandas (giant and red pandas), confirming that food factors drove the convergent evolution of gut microbiota of pandas (18). Therefore, it remains controversial whether the gut microbiota of giant pandas can adapt to their highly specialized diet (14–16). Indeed, studies on the adaptation of gut microbiota of giant pandas to the bamboo diet have mainly focused on captive populations. Considering the obvious difference in gut microbiota composition between captive and wild giant pandas (13, 19, 20), more studies should be performed to uncover mechanisms behind the adaptation of gut microbiota of wild giant pandas to the bamboo diet.

Bamboo is mainly composed of lignin, cellulose, and hemicellulose (21). Lignin attaches to cellulose and hemicellulose in the cell wall and protects them from degradation. Therefore, the gut microbiota of giant pandas needs to first oxidize and decompose lignin before digesting the other components of the bamboo diet. To date, the precise mechanism of lignin degradation by giant pandas is unclear. *Perenniporia medulla-panis* is a fungus found in the gut of giant pandas and exhibits lignin peroxidase activity (22). *Lac51* is a gene-encoding laccase (Lac), an enzyme that can degrade a variety of lignin monomer phenols via oxidation. The sequence similarity alignment of *Lac51* cloned from the fecal microbiotas of giant pandas is closely related to multicopper oxidase gene of *Pseudomonas* sp. (23). However, only a few studies have assessed the potential role of gut microbiota of giant pandas in lignin degradation (17). The community structure of microbiota in wild giant pandas is significantly different from that of captive giant pandas (19). Specifically, *Streptococcus* and *Enterobateriaceae* are the most abundant genera in captive pandas (16, 19, 24), while *Pseudomonas* is the most dominant genus in the gut of wild pandas (19, 20, 25). Similarly, *Pseudomonas* is the most dominant bacteria in the gut of the wild population of red pandas that feed on bamboo (26). Most *Streptococcus* genus and *Enterobateriaceae* family have been reported as primarily pathogens or opportunistic pathogens (27–29). *Pseudomonas* is one of the few bacteria that can efficiently degrade cellulose and lignin (30, 31). For instance, *Pseudomonas* sp. strain ys-1p, *Pseudomonas fluorescens*, and *Pseudomonas* PKE117 can degrade several lignin monomers and dimers (32–35). Davinia et al. revealed that *Pseudomonas putida* KT2440 secretes vesicles carrying enzymes that can degrade lignin and its derivatives (36). Whether the *Pseudomonas*-associated bacteria in wild giant pandas can potentially degrade lignin is still unclear.

A recent study on the adaptation of gut microbiome of giant pandas to different diets and habitats before and after reintroduction identified four genes encoding enzymes, including catalase peroxidase, dioxygenase, quinone reductase, and triacylglycerol lipase, in the metagenome of fecal microbiotas of the giant pandas (37). *Pseudomonas*, *Enterococcus,* and *Lactococcus* are the bacteria enriching these genes. The abundance of the three bacterial genera significantly increased after the release of giant pandas into the wild (37). Yao et al. (20) and Tang et al. (25) also reported that *Pseudomonas* abundance in the gut of captive giant pandas significantly increased after their release into the wild, suggesting that the diversity of gut microbiota of giant pandas increased after reintroduction in the wild. Notably, *Pseudomonas* is the main cyanide-degrading bacteria in the gut of bamboo-eating pandas (wild giant and red pandas), revealing their adaptation to the bamboo diet (38). Collectively, these studies suggest that *Pseudomonas* is the main bacteria responsible for the adaptation of giant pandas to the bamboo diet. However, it is still not clear whether the strain of *Pseudomonas* in the gut of giant pandas can degrade lignin. Also, the specific metabolic pathway of degrading lignin has not been elucidated. In this study, the potential role of the gut microbiome of giant pandas in lignin degradation was explored via metagenomic sequencing to reveal the metabolic function of gut microbiota involved in lignin degradation in bamboo-eating pandas (giant and red pandas), herbivorous, omnivorous, and carnivorous. High-quality individual draft genomes (bins) were produced based on the shotgun metagenomic assemblies. The genomes were then used to reveal the metabolic pathways related to lignin degradation. In addition, *Pseudomonas*-associated strain were isolated from the feces of giant pandas. Lignin degradation and extracellular secretion ability of ligninolytic enzymes were also verified *in vitro*.

This study reveals the specific metabolic pathway of lignin degradation in giant pandas and provides new evidence for adaptive evolution of giant pandas to the bamboo diet. Therefore, these findings provide a basis for future studies on giant pandas, which is important for conservation.

## MATERIALS AND METHODS

### Sample collection and individual information

Fecal samples were collected from both wild and captive animals. Fresh fecal samples were collected from wild giant pandas (*Ailuropoda melanoleuca*) (*n* = 7) and red pandas (*Ailurus fulgens*) (*n* = 5) in Fengtongzhai Nature Reserve (Ya’an, Sichuan Province, China). In addition, fecal samples from captive giant pandas (*n* = 7) and red panda (*n* = 5) were collected from the China Conservation and Research Center and Bifengxia Ecological Zoo (Ya’an, Sichuan Province, China), respectively. The feces were aseptically collected by ranger staff as part of their daily monitoring. We assessed the freshness of the feces by considering both color and surface sheen. After the contaminated part of the surface is removed, the samples were immediately frozen in liquid nitrogen, then transferred to −80°C refrigerator for further utilization.

Previous studies showed that the seven fecal samples from wild giant pandas were from seven different animals (19, 39). The five wild red panda samples have also been confirmed to be from different individuals via GPS collars. The detailed sample information, including age, gender, and population, is shown in Table S1.

### DNA extraction and sequencing

Total microbial genomic DNA was extracted from the fecal samples using the PowerFecal DNA isolation kit (QIAGEN, Inc., Valencia, CA, USA), according to the manufacturer’s instructions. The DNA concentration and purity were measured using the Qubit (Thermo Fisher Scientific, Waltham, MA, USA). Agarose gel electrophoresis was performed to assess the DNA quality. The DNA samples that met the criteria of metagenomic sequencing were used for library preparation: (i) DNA concentration of >15 ng/µL; (ii) total amount of DNA >6 µg; (iii) non-contaminated and intact DNA fragment.

Shotgun metagenomic DNA libraries were constructed based on the Illumina TruSeq DNA Sample Prep V2 Guide (Illumina, Inc.; San Diego, CA, USA), with shearing to 300- to 400-bp fragments. Shotgun metagenomic sequencing was performed on Illumina platform using paired-end 2 × 150 bp chemistry (Novogene, Beijing, China). Ultimately, we obtained gut metagenome data via shotgun metagenomic sequencing from seven wild giant pandas, five wild red pandas, seven captive giant pandas, and five captive red pandas.

### Downloading of gut metagenomic data

The composition of gut microbiotas is significantly different between captive and wild animals. In this study, only data sets from wild mammals were included for joint analysis, except for bamboo-eating animals. Meanwhile, only the metagenome data generated by Illumina platform were downloaded to avoid the possible bias caused by different sequencing platforms. Gut metagenomics data of six red pandas and eight giant pandas from the study of Zhu et al. (38) were retrieved from the National Centre for Biotechnology Information’s Sequence Read Archive (38). The previously published mammalian metagenome data set representing herbivores (*n* = 97), omnivores (*n* = 10), and carnivores (*n* = 14) were downloaded from Levin et al.’s study (40). In addition, the published data of tiger (*n* = 6) (41), black rhinoceros (*n* = 17) (42), David’s deer (*n* = 30) (38), *Gazella subgutturosa* (*n* = 4) (43), Chinese pangolin (*n* = 5), and Malayan pangolin (*n* = 5) were also downloaded from the Genome Sequence Archive (44). The published raw metagenome sequences used in this study were showed in Table S2.

### Shotgun metagenomic sequence analysis

Adapter sequences were removed from all the sequence data using Cutadapt v1.9.1 software (45). Raw paired-end reads were processed by Trimmomatic software to filter out low-quality sequences using a sliding window (5-bp bases) (46). The criteria for quality control were as follows: (i) a sequence was removed once its average quality within the window fell below Q20; (ii) sequences containing any N-bases were filtered out; (iii) reads that were below 50 bp in length were dropped; (iv) only paired-end reads were retained. Animal feces often contain cells shed from their intestines. Therefore, total bacterial DNA extracted from animal feces usually contains the DNA of the host, indicating that the metagenomic sequencing data may contain part of the host genome sequences. After quality control, Bowtie2 (47) was employed to blast the data set of each species to their genome sequences. Sequences with >90% similarity to the genome sequences of the host were removed.

High-quality reads were assembled into contigs using metaSPAdes with the default parameters (48). Assembled contigs with more than 500 bp in length were retained for subsequent analyses. Open reading frames (ORFs) of gut microbiome of each species were predicted from the assembled contigs using MetaGeneMark ORFs ark v 2.8 (49). The non-redundant gut microbial gene set of each species was generated with a 90% identity cutoff using CD-HIT v4.8.1 (50). Data were randomly sub-sampled to the minimum number of sequences in all samples using seqtk-master(https://github.com/lh3/seqtk) before aligning the sequences against the non-redundant panda gut microbiome gene set to reduce biases caused by different sequence depths. Bowtie2 (47) was used to align the paired-end clean reads of each sample against their non-redundant gut microbial gene set. Gene abundance *A*(*g*) in each sample was determined according to Qin et al. (51) as follows:


$$A(g)=\frac{N\left(g\right)}{L\left(g\right)}$$

where “*N*” denotes the number of paired-end clean reads mapped to a gene, *“L*” denotes gene length, and “g“” denotes gene.

The relative abundance of each gene in each sample (*RA*(*g*)) was calculated as follows:


$$RA(g)=\frac{N(g)}{L(g)}*\frac{1}{\sum_{i=1}^{n}\frac{Ni}{Li}}$$

Non-redundant gut microbial gene set of each species was aligned against the Kyoto Encyclopedia of Genes and Genomes (KEGG) online database to annotate the gene functions (52). GHOSTX searches were selected as the assignment method, and other parameters were set as default (53).

Kraken2 was used for taxonomic classification on the clean reads to explore the relative abundance of *Pseudomonas*-associated bacteria in the gut of bamboo-eating pandas and other mammals.

### Extraction of individual draft genomes (bins) from metagenomic data of wild giant pandas

High-quality individual draft genomes (bins) of gut bacteria of wild giant pandas were obtained from shotgun metagenomic assemblies using MetaWRAP (v1.2) to explore the gut strain of giant pandas with the potential to degrade lignin (54). Metagenomic assemblies were binned into draft genomes using the binning module in three metagenomic binning software (MaxBin2, metaBAT2, and Concoct). All bins recovered from the assembly were combined to obtain a single complete bin set using Bin_refinement module. Furthermore, Reassemble_bins module was used to improve N50, completion, and reduce contamination of the bins. Reassembled bins with completeness >70% and contamination <5% were considered as high-quality individual draft genomes (bins) for subsequent analysis. Estimation of bin abundances across samples was performed using the metaWRAP-Quant_bins module. Bins were uploaded to RAST (Rapid Annotations using Subsystems Technology) web server for the identification of species and potential functions of the genome. All parameter options were set at default, and the closest neighbors of bins (with the highest “Identify Score”) were considered as the possible species. A phylogenetic tree was constructed based on all bins using PhyloPhlAn 3.0 tool (55). Furthermore, 40 complete *Pseudomonas* genomes representing the four closest neighbors of *Pseudomonas*-associated strains recovered by binning were downloaded for phylogenetic analysis.

### Laccase-like multicopper oxidase gene cloning and sequencing in the feces of giant pandas

Laccase-like multicopper oxidase gene in the feces of wild giant pandas was cloned and sequenced to explore the presence of laccase gene that can degrade lignin into derivatives in the gut microbiotas in giant pandas. Three fresh fecal samples of wild giant pandas were used for the extraction of genomic DNA. Total genomic DNA of microbiotas was extracted from feces as described above . Laccase-like multicopper oxidase gene short fragments (cbr1- cbr2) were amplified via PCR using the degenerate primers Cu1AF (5′-ACM WCBGTY CAY TGG CAY GG-3′) and Cu2R (5′-G RCT GTGGTA CCA GAA NGT NCC-3′) (56). The PCR volume was 25 µL comprising 2.5-µL 10× ExTaq Buffer, 2.0-µL dNTP mixture (5 mM each), 1-µL forward primer (10 µM), 1-µL reverse primer (10 µM), 0.5-µL Taq DNA polymerase (10 U/µL), 1.0-µL DNA template (25 ng), and 17.0 µL ddH_2_O. Thermocycling parameters included an initial denaturing step at 95°C for 3 min, 30 cycles of 95°C for 30 s, 55°C for 30 s, and 72°C for 60 s and a final extension at 72°C for 5 min.

PCR products were detected using 2.0% agarose gel electrophoresis, and fragments (~150 bp) were obtained for subsequent analysis. Bands with the expected fragment size were purified using Axygen Gel Extraction Kit (Axygen, Silicon Valley, USA) according to the manufacturer’s instructions. The purified target fragment was ligated into the pMD19-T vector system (TaKaRa, Dalian, China) and transformed in *Escherichia coli* DH5α cell according to the manufacturer’s instructions. A total of 50–85 positive clones per fecal sample were selected. The size of the inserted bacterial laccase-like gene fragment was evaluated via PCR, and sequencing was performed by Beijing Genomics Institute (BGI, Shenzhen, China) using primer RV-M. Nucleotide sequences were manually proofread, and similar sequences were obtained through BLAST search in GenBank. Nucleotide sequences were translated into amino acid sequences and were aligned using ClustalW program (57). Sequence analysis and phylogenetic reconstruction were performed using Mega 11 software (58). The neighbor-joining tree was reconstructed based on the maximum composite likelihood method. The strains encoding laccase-like multicopper oxidase enzyme were identified as different species.

### Isolation and identification of *Pseudomonas*-associated bacteria

*Pseudomonas*-associated bacteria were screened in fresh fecal samples of wild giant pandas. Briefly, 1-g fecal sample was dissolved in 100-mL sterile saline solution to form a fecal solution. The fecal solution was diluted 10^−7^ times using sterile saline solution for single colony plotting on an agar plate. The diluted fecal solution (200 µL) was inoculated into *Pseudomonas*-associated bacteria solid screening medium containing 16.0 g/L gelatin peptone, 10.0 g/L acid hydrolyzed casein, 10.0 g/L K₂SO₄, 1.4 g/L Mg_2_Cl, 0.2 g/L cetyltrimethylammonium bromide, 10 mL/L glycerol, 15 mg/L nalidixic acid, and 12.0 g/L agar. Single colonies were obtained after incubating the sample at 37°C for 24 h. The single colonies were re-streaked thrice to obtain a pure colony for strain identification.

Single colonies were incubated with Luria broth (LB) liquid medium at 37°C, 200 rpm for 24 h. The LB liquid medium was then centrifuged at 12,000 × *g* for 5 min, and the supernatant was discarded. DNA isolation kit (Tiangen Biotech, Beijing, China) was used to extract genomic DNA of the bacterial cells following the manufacturer’s instructions. The 16S rDNA sequence was amplified with bacterial universal primers 27F (AGAGTTTGATCCTGGCTCAG) and 1492R (GGTTACCTTGTTACGACTT). The amplified fragment was sequenced by Tsingke Biotechnology Co., Ltd. The obtained sequences were compared with the NCBI sequences using the BLAST website.

### Determination of lignin-degrading capability of *Pseudomonas*-associated bacteria

The isolated *Pseudomonas*-associated strains were inoculated into modified M9 solid medium containing 6.78 g/L Na2HPO4, 3 g/L KH2PO4, 0.5 g/L NaCl, 1 g/L NH4CL, 2 mM MgSO4, 100 µM CaCl2, 100 µM MnSO4, 100 µM CuSO4, 100 µM FeSO4, 50 µM ZnSO4, 2 g/L kraft lignin, and 12.0 g/L agar (with lignin as the sole carbon source; pH 7.0). The cell growth and lignin degradation were determined to test the ligninolytic activity of the *Pseudomonas*-associated strains. *Pseudomonas*-associated strains were inoculated in LB medium and cultured at 37°C and 200 rpm for 24 h while shaking. The culture broth was centrifuged at 12,000 × *g* for 5 min to obtain the bacterial cells. The bacterial cells were inoculated into 100 mL of modified M9 medium (pH 7.0) containing 2 g/L kraft lignin (initial OD600 = 0.1), then cultured at 37°C, 200 rpm for 7 days. Fermentation broth (1 mL) was taken daily to measure the growth curve and lignin-degrading capability of the *Pseudomonas*-associated strains. Cell growth was determined at OD600; then, the growth curve of *Pseudomonas*-associated strains was plotted. The lignin degradation rate was evaluated by monitoring the decrease in A280. Degradation ratio was calculated as follows: $degradation\%=(A_{control}-A_{sample})\;/A_{control}*100\%$. All the experiments were performed in triplicates.

### The decolorization capability for aromatic dyes and enzyme assays

The capability of the screened isolates to decolorize the aromatic dyes with almost similar structures to lignin fragments was determined to further evaluate whether *Pseudomonas*-associated strains can secrete extracellular ligninolytic enzymes to degrade lignin. The *Pseudomonas*-associated strains were inoculated into aniline blue solid medium (pH 7.0) containing 10 g/L glucose, 10 g/L peptone, 0.04 g/L water-soluble aniline blue, and 12 g/L agar. The decolorization circle of the *Pseudomonas*-associated strains on aniline blue solid medium was confirmed; then, the bacterial cells with OD600 = 0.1 were inoculated into 50-mL aniline blue liquid medium (pH 7.0). The samples were cultured at 37°C and 200 rpm for 96 h while shaking. The aniline blue liquid medium without bacteria was used as the blank control. The culture medium was centrifuged at 12,000 rpm for 5 min every 24 h, and the absorbance of supernatants was measured at 600 nm. The decolorization ratio was calculated as follows: $decolorization\%=(A_{control}-A_{sample})\;/A_{control}*100\%$. All the experiments were performed in triplicates.

The *Pseudomonas*-associated strains were inoculated into 100-mL modified M9 medium (pH 7.0) containing 2 g/L bamboo powder at an initial OD600 of 0.1 and then cultured at 37°C and 200 rpm for 7 days while shaking. The culture medium was centrifuged at 12,000 rpm for 2 min every 24 h, and the supernatants were used to determine extracellular ligninolytic enzymes. Lac activity was determined by measuring the oxidation of ABTS [2,2-azino-bis (3-ethylbenzothiazoline-6-sulphonic acid)] to ABTS radical at 420 nm using a reaction mixture (200 µL) containing 180-µL ABTS (0.5 mmol/L) (dissolved in 0.1 mmol/L HAc-NaAc buffer solution, pH 4.5) and 20-µL cell-free supernatant. Lignin peroxidase (LiP) was evaluated by monitoring the oxidation rate of azure B at 651 nm using a reaction mixture (200 µL) containing 180-µL azure B (0.5 mmol/L) (dissolved in 0.1 mmol/L HAc-NaAc buffer solution, pH 4.5) and 20-µL cell-free supernatant. Mn-peroxidase (MnP) activity was evaluated by monitoring the oxidation of 2,6-DMP (2,6-dimethyl phenol) to coerulignone at 469 nm using a 200-µL reaction mixture containing 180-µL 2,6-DMP (0.5 mmol/L) (dissolved in 0.1 mmol/L HAc-NaAc and MnSO4 buffer solution, pH 4.5) and 20-µL cell-free supernatant. One unit of enzyme activity was defined as the amount of enzyme required to oxidize 1 µmol substrate per minute. All fermentation tests were performed in triplicate.

### Determination of degradation products of lignin by *Pseudomonas*-associated strains

The centrifugal supernatants of lignin fermentation medium of *Pseudomonas*-associated strains were filtered using a 0.2-µm filter membrane, and quality control samples were prepared. Metabolites of the supernatants were extracted before liquid chromatography–mass spectrometry (LC–MS) detection, as previously described (59). Each sample was run in triplicate, and the stability of mass spectrometry was evaluated. LC-MS detection was performed as previously described (60, 61). The raw data were first converted to mzXML format via MSConvert in ProteoWizard software package (v3.0.8789) (62) and processed using XCMS (63) for feature detection, retention time correction, and alignment. The metabolites were identified via accuracy mass (<30ppm) and LC-MS data, which were matched with Human Metabolome Database (http://www.hmdb.ca/) (64), MassBank (http://www.massbank.jp/ (65), LipidMaps (https://www.lipidmaps.org) (66), mzcloud (https://www.mzcloud.org) (67), and KEGG (https://www.kegg.jp) (68). The robust LOESS signal correction (QC-RLSC) was applied for data normalization to correct any systematic bias (69). Ion peaks with relative standard deviations less than 30% in QC were kept ensuring proper metabolite identification. Relative quantification was estimated based on the ratio of the respective peak areas to the total peak areas.

### Statistical analysis

Significant differences in the relative abundance of genes between different diet groups were determined using the Kruskal-Wallis test followed by Dunn’s multiple-comparison post-hoc test. All statistical analyses were performed using GraphPad Prism 7 software (GraphPad Software, Inc., USA). Figures were generated using “pheatmap” packages (70) and different functions (“boxplot,” “barplot,” “pie,” and “plot”) in R base (version 3.6.1) (71). Multivariate statistical analysis and modeling were conducted using the resultant data sets from LC-MS procedures via the Ropls software (72). Models were built based on principal component analysis (PCA) to explore data set variations. The *P*-value and variable importance projection (VIP) produced by OPLS-DA were applied to discover the significance of metabolites (*P* value <0.05 and VIP values >1) in different groups.

## RESULTS

### Metagenome sequencing data

A total of 319,158,629 paired-end metagenome reads were generated in this study. In addition, 5,347,754,253 published raw paired-end reads from 209 wild individual samples (representing bamboo-eating pandas, herbivores, omnivores, carnivores) were retrieved from the public database for comprehensive analysis. A total of 5,117,016,424 clean paired-end reads were retained for subsequent analysis after filtering out the low-quality reads (Q < 20) and host sequences. The final contigs of each species were obtained through *de novo* assembly. Non-redundant gut microbiome gene set of each species was created from contigs.

### Composition gut microbiotas of bamboo-eating pandas

Principle coordinate analysis of predicted metagenomic function (KO genes) based on Bray-Curtis distance showed that wild bamboo-eating pandas formed distinct clusters from other animals (Adonis *R*^2^ = 0.473; *P*-value = 0.001), implying that they have a distinct function (Fig. S1). Moreover, wild bamboo-eating pandas harbored a distinct gut microbiome from captive bamboo-eating pandas. Furthermore, gut microbiome in carnivores was distinct from that of herbivores and omnivores. Overall, the functions of the gut microbiome of different animals clustered according to their food habits, indicating their various roles. Analysis at the genus level showed that *Pseudomonas* and *Enterobacteriaceae* were the most abundant genus in the gut of wild giant pandas (Fig. S2A) and captive giant pandas (Fig. S2B), respectively.

### Metabolic pathways related to lignin degradation in gut microbiotas of giant pandas

**Fig 1:**
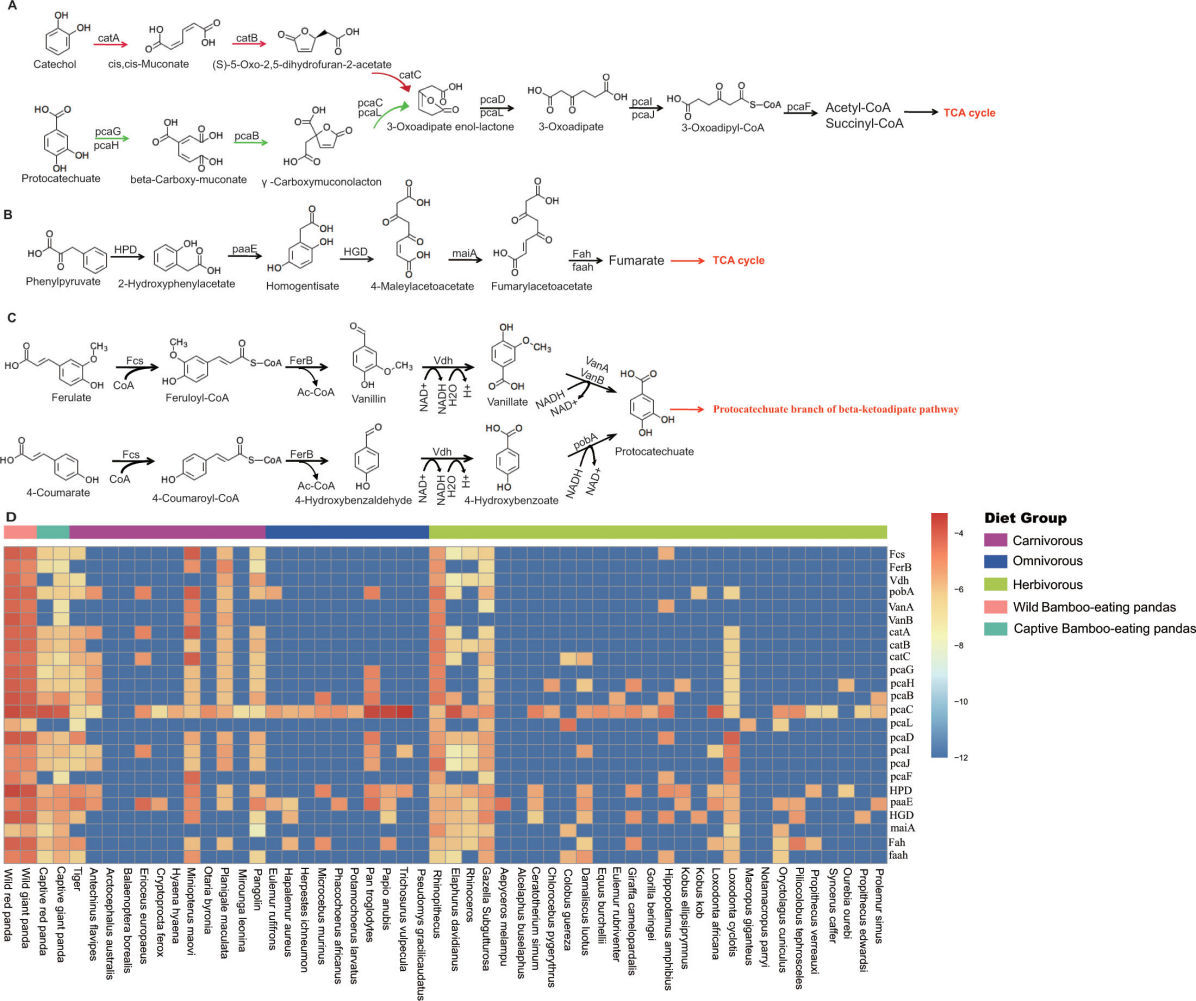
Metabolic pathways involved in lignin derivative degradation enriched in the gut microbiome of wild giant pandas. (A) Beta-ketoadipate pathway, red arrows represent the catechol branch of beta-ketoadipate pathway, green arrows represent the protocatechuate branch of beta-ketoadipate pathway, and black arrows represent parts of the metabolic pathway shared by catechol and protocatechuate branch. (B) Homogentisate pathway for degradation of aromatic compounds. (C) The metabolic pathway for catabolism of lignin monomers *p*-coumarate and ferulate into protocatechuate, which can be further degraded into acetyl-CoA and succinyl-CoA through protocatechuate branch of beta-ketoadipate pathway. (D) Average abundance of genes coding for enzymes implicated in the beta-ketoadipate pathway, homogentisate pathway, and *p*-coumarate and ferulate degradation pathway in the gut of bamboo-eating pandas, terrestrial mammals, and wood-feeding insects presented as a heat map (log_10_ abundance).

Pathway analysis based on the genome of microbiotas derived from feces of wild bamboo-eating pandas (giant and red pandas) showed enrichment of the complete metabolic pathway of central aromatic intermediates, including catechol branch of beta-ketoadipate pathway (Cbβ-KAP) (Fig. 1A), protocatechuate branch of beta-ketoadipate pathway (Pbβ-KAP) (Fig. 1A), and homogentisate pathway of aromatic compound degradation (Fig. 1B). Notably, 4-hydroxyphenylpyruvate dioxygenase (HPD), ring-1, 2-phenylacetyl-CoA epoxidase subunit (paaE) homogentisate 1, 2-dioxygenase (HGD), maleylacetoacetate isomerase (maiA), fumarylacetoacetase (Fah), and fumarylacetoacetate hydrolase (faaH) catalyzed reactions in homogentisate pathway (Fig. 1B). Catechol-1, 2-dioxygenase (catA), muconate lactonizing enzyme (catB), and muconolactone isomerase (catC), involved in catalysis of degradation reactions in Cbβ-KAP, were significantly enriched (Fig. 1A). Pbβ-KAP was metabolized through protocatechuate 3,4-dioxygenase alpha chain (pcaG), protocatechuate 3,4-dioxygenase beta chain (pcaH), 3-carboxy-cis, cis-muconate cycloisomerase (pcaB), 4-carboxymuconolactone decarboxylase (pcaC, pcaL), and 3-oxoadipyl-CoA thiolase (pcaF). Protocatechuate and catechol are degraded to 3-oxoadipate enol-lactone and share the same metabolic pathway. The degradation process is catalyzed by beta-ketoadipate enol-lactone hydrolase (pcaD, pcaL), 3-oxoadipate CoA-transferase subunit A (pcaI), and 3-oxoadipate CoA-transferase subunit B (pcaJ) (Fig. 1A). The final product of Cbβ-KAP and Pbβ-KAP degradation is succinyl-CoA and acetyl-CoA, whereas phenylpyruvate is eventually degraded to fumarate through the homogentisate pathway. Succinyl-CoA, acetyl-CoA, and fumarate can join the tricarboxylic acid (TCA) cycle for biosynthesis of valuable products.

Lignin monomers, including ferulate and *p*-coumarate, are finally degraded to protocatechuate through a series of metabolic reactions. Protocatechuate is further degraded to succinyl-CoA through Pbβ-KAP (Fig. 1C). Degradation of ferulate is catalyzed by feruloyl-CoA synthase (Fcs), feruloyl-CoA hydratase (FerB), vanillin dehydrogenase (Vdh), vanillate monooxygenase (VanA), and vanillate monooxygenase ferredoxin subunit (VanB). Fcs, FerB, Vdh, and p-hydroxybenzoate 3-monooxygenase (pobA) catalyze degradation reactions of *p*-coumarate. These genes were identified in the genome of microbiotas derived from the feces of wild giant pandas.

Taxonomic assignment of enzymes revealed that a large proportion (26.08% on average) of the predicted genes were present in species within the *Pseudomonas* genus bacteria. For example, 8 out of 12 vdh, 16 out of 54 vanA, and 38 out of 163 pcaD genes in this study were homologous to genes from *Pseudomonas*-associated bacteria (Table S3). This finding indicated that predicted genes coding for lignin derivatives or monomer-digesting enzymes were mainly contributed by *Pseudomonas* genus.

**Fig 2:**
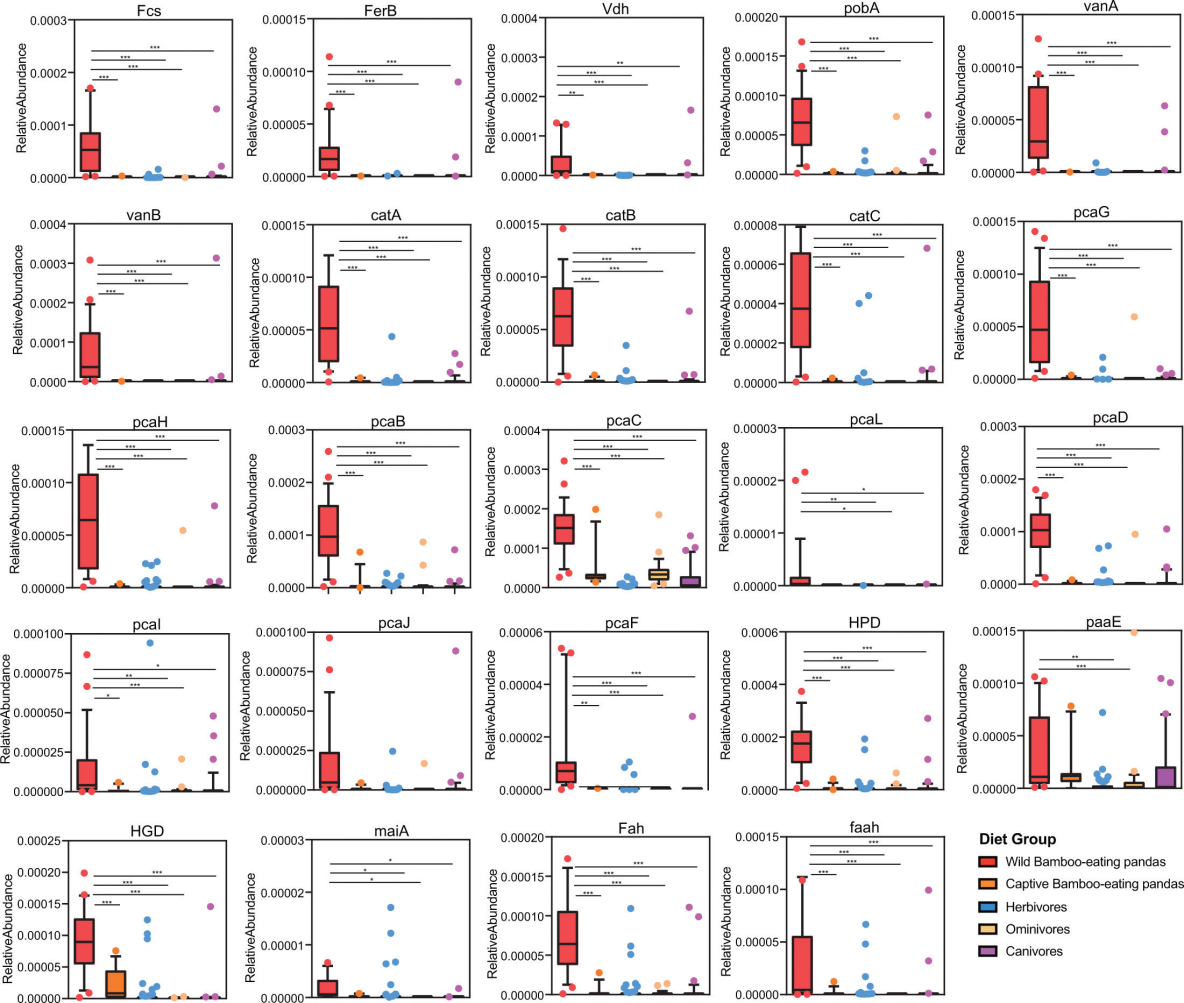
Relative abundance of genes encoding enzymes for beta-ketoadipate pathway, homogentisate pathway, *p*-coumarate, and ferulate degradation pathway in the gut of bamboo-eating pandas, terrestrial mammals, and wood-feeding insects presented as box plots (*P* values were calculated by the Kruskal-Wallis test followed by Dunn’s multiple-comparison post-hoc test: *P < 0.05, **P < 0.01, and ***P < 0.001).

Heatmap analysis showed that the average abundance of genes involved in the metabolism of central aromatic intermediates, *p*-coumarate, and ferulate was highest in wild bamboo-eating pandas compared with the abundance in other animals (carnivores, omnivores, herbivores, and captive bamboo-eating pandas) (Fig. 1D). The relative abundance of these genes was significantly higher in wild bamboo-eating pandas than in other animals (Fig. 2).

### Individual draft genomes (bins) of gut microbiotas from wild giant pandas

**Fig 3:**
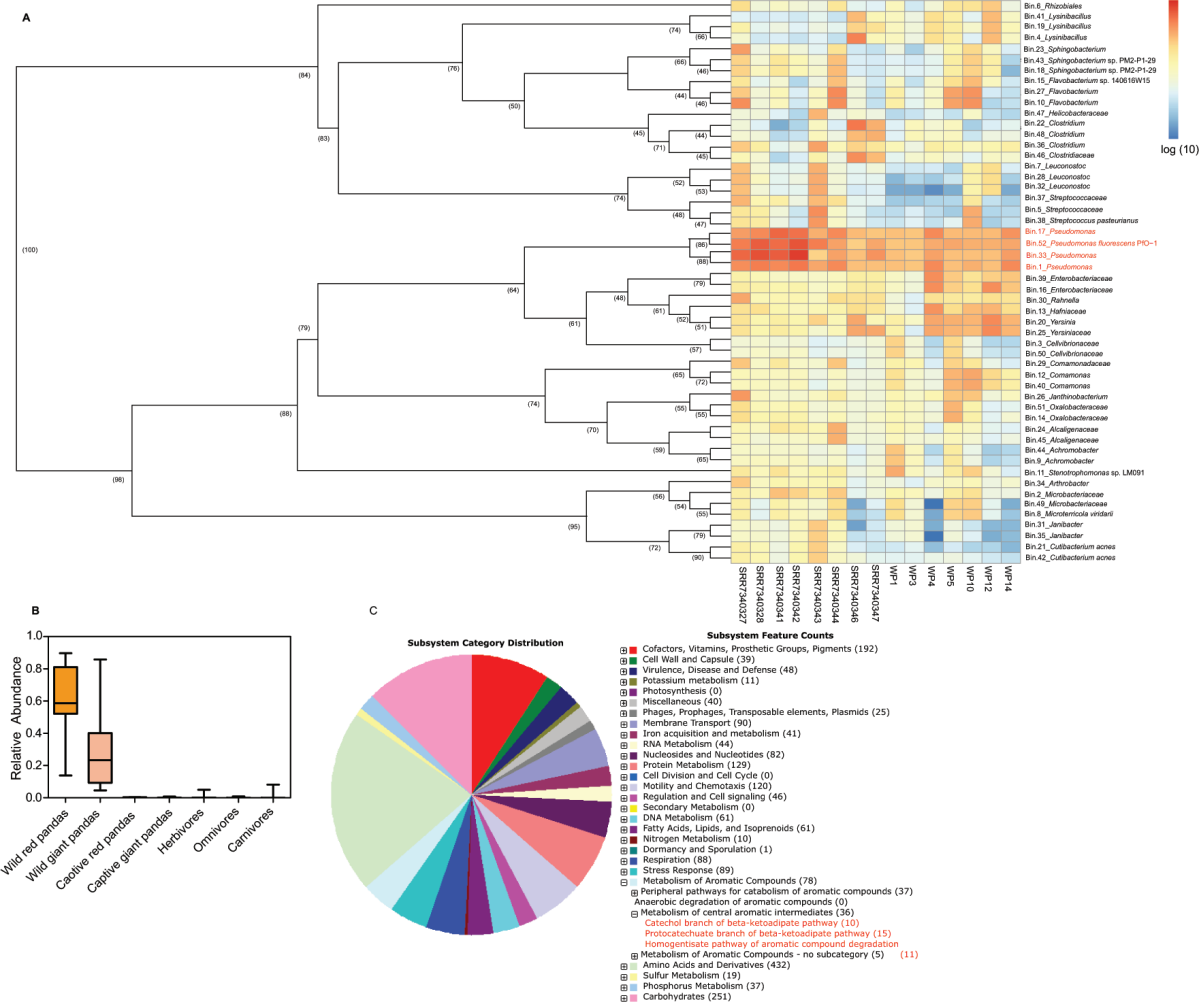
(**A**) Phylogenetic analysis of the high-quality strain draft genomes (bins) from metagenome sequencing and the relative abundance of these strains in the gut of wild giant pandas. The left panel shows the neighbor-joining tree using high-quality strain draft genomes (bins) obtained from metagenome sequencing. The numbers in parentheses on the phylogenetic tree represent the bootstrap value of the nodes. The right panel represents the average abundance of each strain draft genomes (bins) in the gut of wild giant pandas. (**B**) Functional annotation of the genome of *Pseudomonas*-associated bin (bin 1, bin 17, bin 33, and bin 52) in the gut of wild giant panda. Annotation results of the four genomes were similar; thus, one bin (bin 1) was selected to present the functional annotation results.

Fifty-two high-quality individual draft genomes (bins) were obtained from gut metagenomic data of wild giant pandas (Fig. 3A; Table S4). The average abundances of Bin 1, Bin 17, Bin 33, and Bin 52 were the highest in the gut of wild giant pandas (Fig. 3A). Bin 1, Bin 17, and Bin 33 were classified as *Pseudomonas*, whereas Bin 52 was identified as *Pseudomonas fluorescens* according to the principle of the closest strain annotation. Further comparison showed that the relative abundance of *Pseudomonas*-associated bacteria in the gut was significantly higher in wild giant panda (mean ± SD: 0.35.80 ± 0.22.70) and wild red panda (0.58.34 ± 0.26.52) than in captive red pandas (0.0014 ± 0.0013), captive giant pandas (0.0016 ± 0.0024), herbivores (0.0013 ± 0.0003), omnivores (0.0004 ± 0.0001), and carnivores (0.0005 ± 0.0004) (Fig. 3B).

The draft genome of *Pseudomonas*-associated (bin 1, bin 17, bin 33, and bin 52) (Table S5) and *Achromobacter*-associated (bin 9 and bin 44) strains encoded all genes implicated in Cbβ-KAP and Pbβ-KAP metabolism and homogentisate pathway (Fig. 3C; Table 1). The genome of *Oxalobacteraceae* (bin 14 and bin 51) comprised all genes implicated in Cbβ-KAP and Pbβ-KAP metabolism, whereas the genome of *Janthinobacterium* (bin 26) encoded all genes involved in Cbβ-KAP metabolism and homogentisate pathway (Table 1). In addition, the genome of *Comamonadaceae* (bin 29) and *Alcaligenaceae* (bin 45) encoded all genes implicated in Cbβ-KAP metabolism, whereas the genome of *Flavobacterium* (bin 10 and bin 27), *Stenotrophomonas* sp. LM091 (bin 11), and *Flavobacterium* sp. 140616W15 (bin 15) encoded all genes involved in homogentisate pathway (Table 1). pobA, vanA, vanB, and vdh genes were identified in the genome of *Pseudomonas*-associated Bin (Table S5).

**TABLE 1:** Species and potential functions identification of individual bacteria draft genomes in wild giant pandas

Binning ID	Closest genome name	Catechol branch of β-ketoadipate pathway	Protocatechuate branch of β-ketoadipate pathway	Homogentisate pathway of aromatic compound degradation
Bin 1	*Pseudomonas*	catA, catB, catC	pcaG, pcaH, pcaB, pcaC, pcaL, pcaD, pcaI, pcaJ	HPD, HGD, maiA, Fah, faah, paaE
Bin 2	*Microbacteriaceae*	NA^*a*^	NA	NA
Bin 3	*Cellvibrionaceae*	NA	NA	NA
Bin 4	*Lysinibacillus*	NA	NA	NA
Bin 5	*Streptococcaceae*	NA	NA	NA
Bin 6	*Rhizobiales*	NA	NA	NA
Bin 7	*Leuconostoc*	NA	NA	NA
Bin 8	*Microterricola viridarii*	NA	NA	NA
Bin 9	*Achromobacter*	catA, catB, catC	pcaG, pcaH, pcaB ,pcaC, pcaL, pcaD, pcaI, pcaJ	HPD, HGD, maiA, Fah, faah
Bin 10	*Flavobacterium*	NA	NA	HPD, HGD, maiA, Fah, faah
Bin 11	*Stenotrophomonas* sp. LM091	NA	NA	HPD, HGD, maiA, Fah, faah
Bin 12	*Comamonas*	catB, catC	NA	NA
Bin 13	*Hafniaceae*	NA	NA	NA
Bin 14	*Oxalobacteraceae*	catA, catB, catC	pcaG, pcaH, pcaB ,pcaC, pcaL, pcaD, pcaI, pcaJ	NA
Bin 15	*Flavobacterium* sp. 140616W15	NA	NA	HPD, HGD, maiA, Fah, faah
Bin 16	*Enterobacteriaceae*	NA	NA	NA
Bin 17	*Pseudomonas*	catA, catB, catC	pcaG, pcaH, pcaB, pcaC, pcaL, pcaD, pcaI, pcaJ	HPD, HGD, maiA, Fah, faah
Bin 18	*Sphingobacterium* sp. PM2-P1-29	NA	NA	NA
Bin 19	*Lysinibacillus*	NA	NA	NA
Bin 20	*Yersinia*	NA	NA	NA
Bin 21	*Cutibacterium acnes*	NA	NA	NA
Bin 22	*Clostridium*	NA	NA	NA
Bin 23	*Sphingobacterium*	NA	NA	HPD, HGD
Bin 24	*Alcaligenaceae*	NA	NA	NA
Bin 25	*Yersiniaceae*	NA	NA	NA
Bin 26	*Janthinobacterium*	catA, catB, catC	NA	HPD, HGD, maiA, Fah, faah
Bin 27	*Flavobacterium*	NA	NA	HPD, HGD, maiA, Fah, faah
Bin 28	*Leuconostoc*	NA	NA	NA
Bin 29	*Comamonadaceae*	catA, catB, catC	NA	NA
Bin 30	*Rahnella*	NA	NA	NA
Bin 31	*Janibacter*	NA	NA	HPD, HGD, Fah
Bin 32	*Leuconostoc*	NA	NA	NA
Bin 33	*Pseudomonas*	catA, catB, catC	pcaG, pcaH, pcaB, pcaC, pcaL, pcaD, pcaI, pcaJ	HPD, HGD, maiA, Fah, faah
Bin 34	*Arthrobacter*	NA	NA	NA
Bin 35	*Janibacter*	NA	NA	HPD, HGD, Fah
Bin 36	*Clostridium*	NA	NA	NA
Bin 37	*Streptococcaceae*	NA	NA	NA
Bin 38	*Streptococcus pasteurianus*	NA	NA	NA
Bin 39	*Enterobacteriaceae*			
Bin 40	*Comamonas*	catB ,catC	NA	NA
Bin 41	*Lysinibacillus*	NA	NA	NA
Bin 42	*Cutibacterium acnes*	NA	NA	NA
Bin 43	*Sphingobacterium* sp. PM2-P1-29	NA	NA	HPD, HGD
Bin 44	*Achromobacter*	catA, catB ,catC	pcaG, pcaH, pcaB ,pcaC, pcaL, pcaD, pcaI, pcaJ	HPD, HGD, maiA, Fah, faah
Bin 45	*Alcaligenaceae*	catA, catB ,catC	NA	NA
Bin 46	*Clostridiaceae*	NA	NA	NA
Bin 47	*Helicobacteraceae*	NA	NA	NA
Bin 48	*Clostridium*	NA	NA	NA
Bin 49	*Microbacteriaceae*	NA	NA	NA
Bin 50	*Cellvibrionaceae*	NA	NA	NA
Bin 51	*Oxalobacteraceae*	catA, catB ,catC	pcaG, pcaH, pcaB, pcaC, pcaL, pcaD, pcaI, pcaJ	NA
Bin 52	*Pseudomonas fluorescens* PfO-1	catA, catB, catC	pcaG, pcaH, pcaB, pcaC, pcaL, pcaD, pcaI, pcaJ	HPD, HGD, maiA, Fah, faah

^*a*^ NA indicates the absence of genes involved in this pathway in the genome.

### Phylogenetic analysis of *Pseudomonas* genomes in wild giant pandas

**Fig 4:**
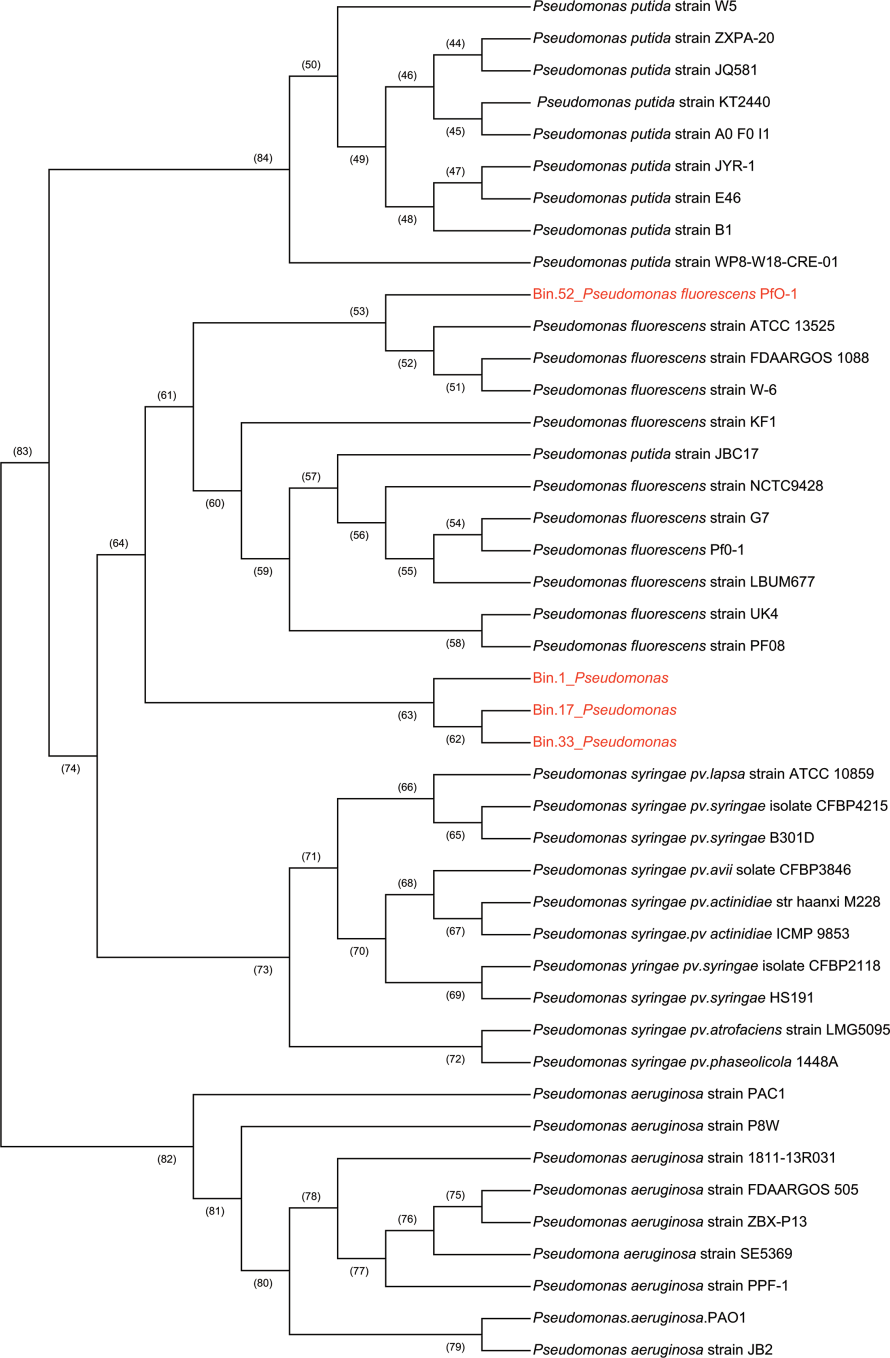
Phylogenetic analysis of *Pseudomonas*-associated genome (bin) in the gut of wild giant panda and the published genomes of *Pseudomonas fluorescens*, *Pseudomonas syringae* pv. *phaseolicola*, *Pseudomonas putida* and *Pseudomonas aeruginosa*. Genomes of 10 different strains for each *Pseudomonas* species were included in the phylogenetic tree (neighbor-joining) analysis. The numbers in parentheses on the phylogenetic tree represent the bootstrap value of the nodes.

The four closest neighbors of bin 1, bin 17, bin 33, and bin 52 were *Pseudomonas fluorescens* PfO-1, *Pseudomonas syringae* pv. *phaseolicola* 1448A, *Pseudomonas putida* KT2440, and *Pseudomonas aeruginosa* PAO1, respectively, as identified through RAST. A total of 40 complete genomes of different strains (10 per species), belonging to *Pseudomonas fluorescens*, *Pseudomonas syringae*, *Pseudomonas putida*, and *Pseudomonas aeruginosa*, were randomly selected to construct phylogenetic trees for further classification of *Pseudomonas* strains in the wild giant pandas. Phylogenetic analysis showed that bin 52 (*Pseudomonas fluorescens*) clustered in the *Pseudomonas fluorescens* clade and that bin 1, bin 17, and bin 33 (*Pseudomonas*) clustered into a single clade in the phylogenetic tree (Fig. 4). Similar to *Pseudomonas* genomes in this study (Fig. 3C), the genome of *Pseudomonas fluorescens* PfO-1 (Fig. S3A), *Pseudomonas syringae* pv. *phaseolicola* 1448A (Fig. S3B), *Pseudomonas putida* KT2440 (Fig. S3C), and *Pseudomonas aeruginosa* PAO1 (Fig. S3D) encoded all enzymes implicated in the metabolism of central aromatic intermediates.

### Laccase-like multicopper oxidase genes were present in the gut of wild giant pandas

Approximately 150-bp fragments between regions I and II of laccase-like multicopper oxidase genes, which are crucial in lignin degradation, were obtained from the DNA derived from feces of wild giant pandas through PCR amplification. A total of 228 colonies from three samples (76 per sample) of wild giant pandas were chosen at random for sequencing. Nucleotide sequences obtained were used as query sequences for BLAST search to obtain the sequences deposited in GenBank nucleotide database. The BLAST results indicated that most (except for colony 2) PCR products highly corresponded to the laccase-like multicopper oxidase genes of bacteria. The results showed that 76% of products of colonies had a high similarity (>90%) to gene sequences retrieved from GenBank nucleotide database. Notably, only two clones had less than 80% similarity compared with nucleotide sequences deposited in the GenBank (Table S6). The most consistent alignment results obtained from GenBank sequences were considered as the possible species of laccase fragments. A total of 228 colony products were identified as *Verrucomicrobiaceae* HC12, *Verrucomicrobiaceae* ONA9, *Pseudomonas antarctica*, *Janthinobacterium svalbardensis*, *Pseudomonas gingeri*, *Agrobacterium*, *Flavobacterium* sp., *Acinetobacter* sp., *Pseudomonas azotoformans*, *Pseudomonas fluorescens*, *Pseudomonas putida*, *Klebsiella pneumonia*, *Stenotrophomonas* sp., *Caulobacter* sp., *Brevundimonas diminuta*, *Brevundimonas vancanneytii*, *Brevundimonas naejangsanensis*, and *Sphingosinicella* sp. (Table S6).

**Fig 5:**
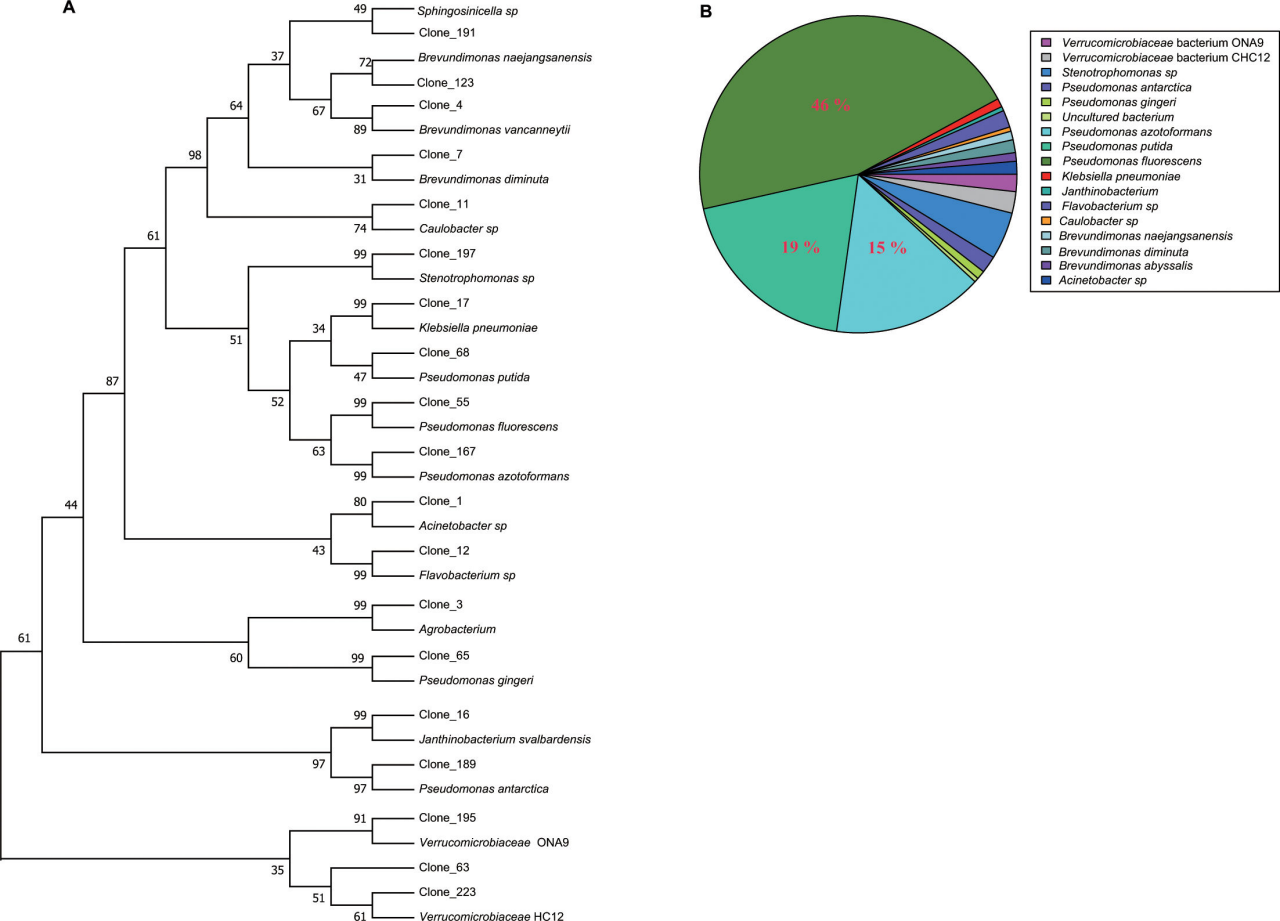
Phylogenetic analysis and composition of bacterial species with laccase-like multicopper oxidase gene derived from the feces of wild giant pandas. (**A**) Neighbor-joining phylogenetic tree constructed using laccase-like multicopper oxidase gene sequences from the feces of wild giant pandas and the most similar alignment sequence obtained from GeneBank. One laccase-like multicopper oxidase gene sequence was randomly selected from each identified bacterial species. The numbers on the phylogenetic tree represent the bootstrap value of the node. (**B**) Pie plots showing the composition of bacterial species of laccase-like multicopper oxidase gene in the feces of wild giant pandas.

Eighteen published laccase-like multicopper oxidase gene sequences were retrieved for phylogenetic analysis. Phylogenetic results indicated that the laccase-like multicopper oxidase genes in the feces of wild giant pandas clustered into 19 clades. Notably, each representative clone fragment showed a close evolutionary relationship with the reference sequences obtained from GenBank (Fig. 5A). *Pseudomonas*-associated sequences were abundant in feces libraries of the wild giant pandas, where they accounted for 83.33% of the total number of sequences (Fig. 5B). The proportion of *Pseudomonas fluorescens*-associated sequences was the highest (about 46%), followed by the abundance of *Pseudomonas putida*- and *Pseudomonas azotoformans*-associated sequences, accounting for 19% and 15%, respectively (Fig. 5B; Table S6). In addition, predicted genes coding for multicopper oxidase enzymes were identified in the genome of *Pseudomonas*-associated bin (Table S5). These results imply that laccase-like multicopper oxidase gene in the gut of giant pandas may be mainly contributed by bacteria in the *Pseudomonas* genus.

### Lignin degradation capability of single *Pseudomonas*-associated bacteria

Three most abundant *Pseudomonas*-associated bacteria were obtained based on colonial morphology difference in the *Pseudomonas*-associated bacteria solid screening medium after three repeated cultivations. These *Pseudomonas*-associated isolates were closely matched to *Pseudomonas putida* strain cqsH1 (99%), *Pseudomonas* sp. strain QW16-14 (99%), and *Pseudomonas oryzihabitans* strain h-2 (99%) based on 16S rRNA gene sequence homology.

**Fig 6:**
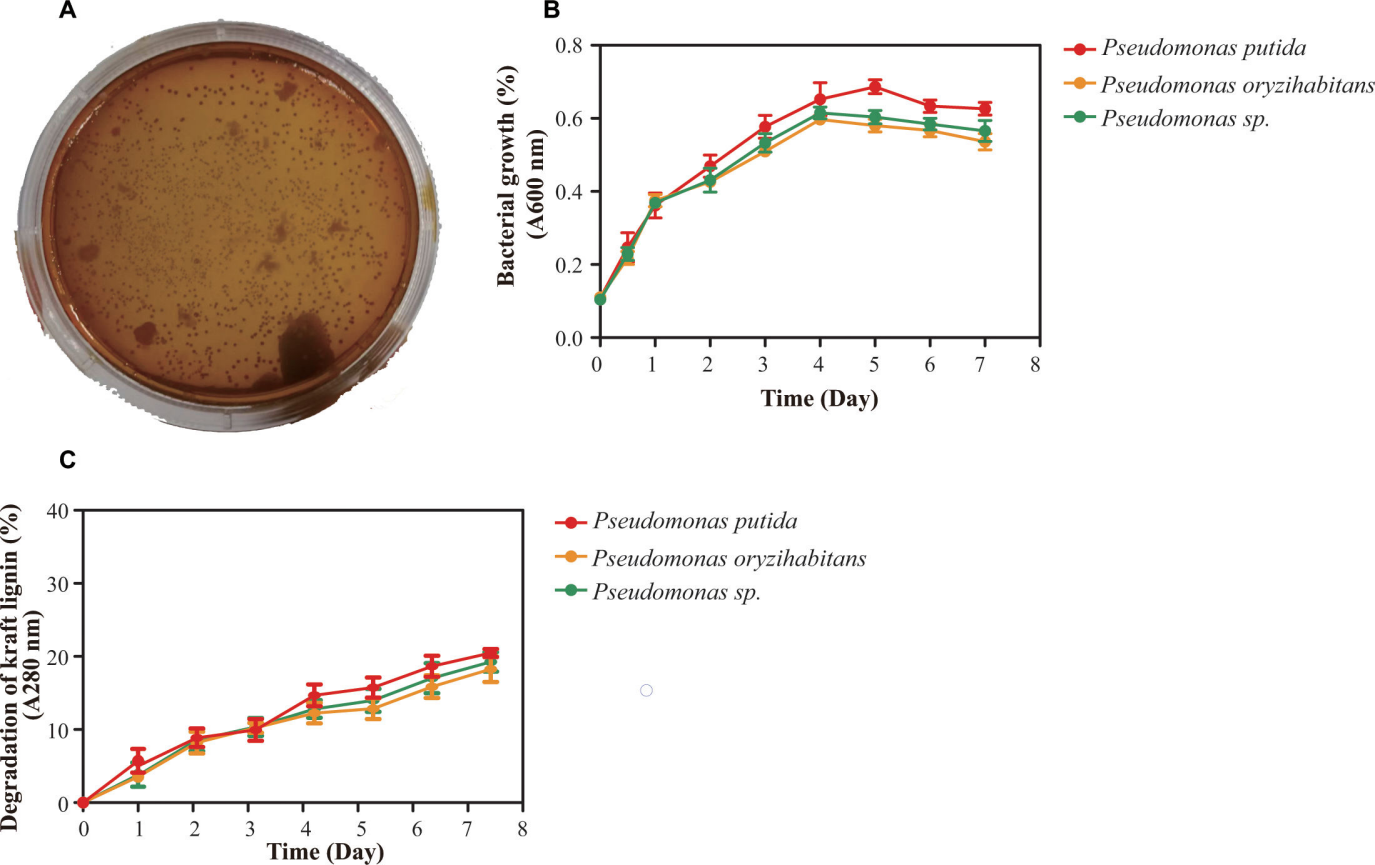
Experimental results of lignin degradation by *Pseudomonas*-associated strain. (**A**) Growth of *Pseudomonas*-associated strain on solid medium with lignin as the sole carbon source. (**B**) The growth curve of *Pseudomonas*-associated strain on liquid medium with lignin as the sole carbon source. (**C**) Lignin degradation curve.

Three *Pseudomonas*-associated isolates grew well on a solid medium with lignin as the sole carbon source (Fig. 6A). The growth and lignin-degrading curves of *Pseudomonas putida*, *Pseudomonas* sp., and *Pseudomonas oryzihabitans* are shown in Fig. 6B. The lag phase, exponential growth phase, stationary phase, and decline phase occurred at 0–12 h, 24–72 h, 84–120 h, and 132–168 h, respectively. The initial average absorbance of the lignin culture medium was 4.47 at 280 nm, which decreased to 3.21 after 7 days of incubation. The degradation rates for *Pseudomonas putida*, *Pseudomonas* sp., and *Pseudomonas oryzihabitans* after 7 days were 19.66%, 19.14%, and 18.17%, respectively (Fig. 6C).

### Extracellular ligninolytic enzymes of *Pseudomonas*-associated isolates

**Fig 7:**
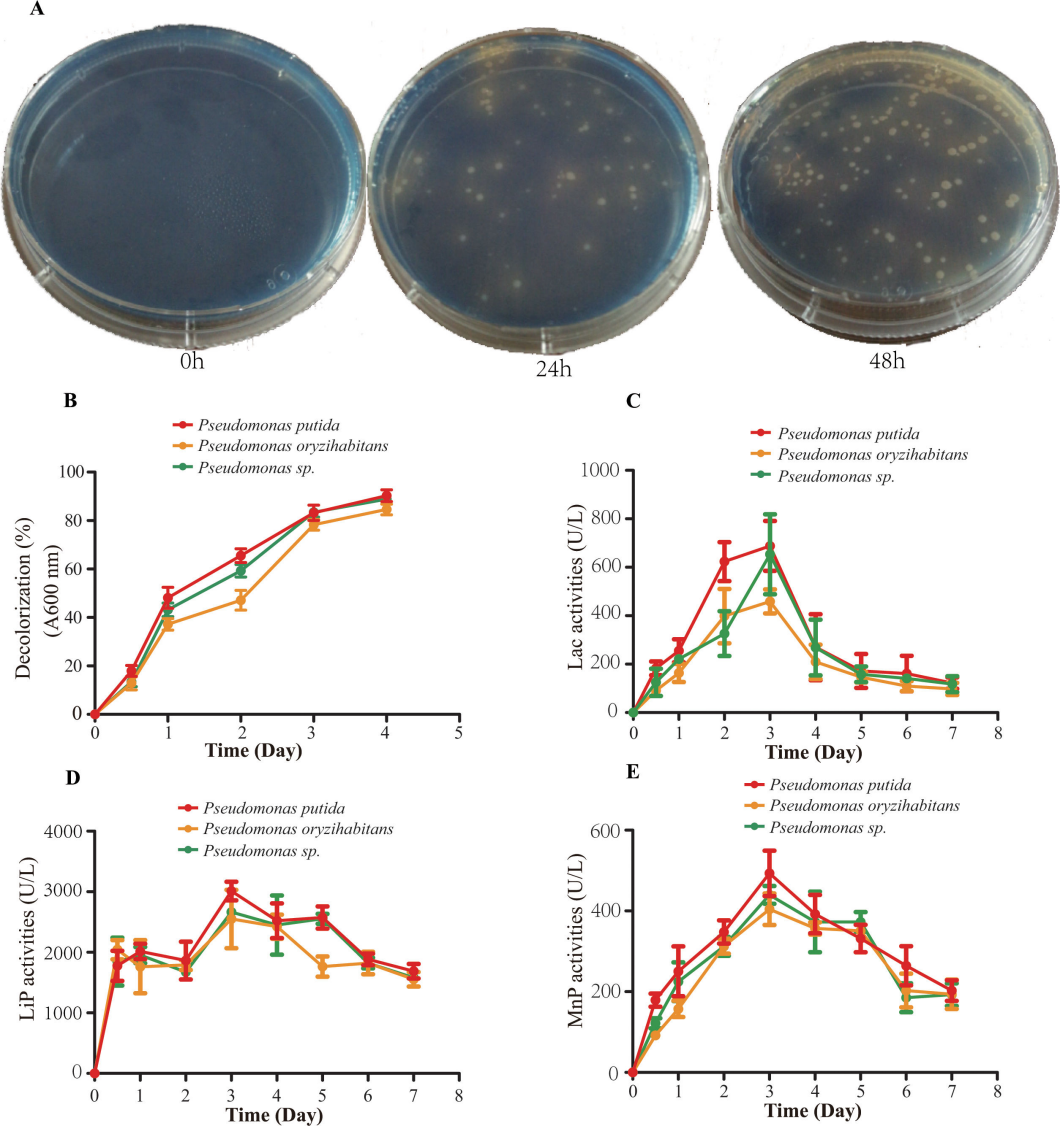
Analysis of extracellular enzyme activity of *Pseudomonas*-associated strain. (**A**) Decolorization efficiency of *Pseudomonas*-associated strain on aniline blue solid medium. (**B**) Decolorization rate curve of aniline blue B. Activities of the Lac (**C**), LiP (**D**), and MnP (**E**) during the incubation of *Pseudomonas*-associated strain.

**Fig 8:**
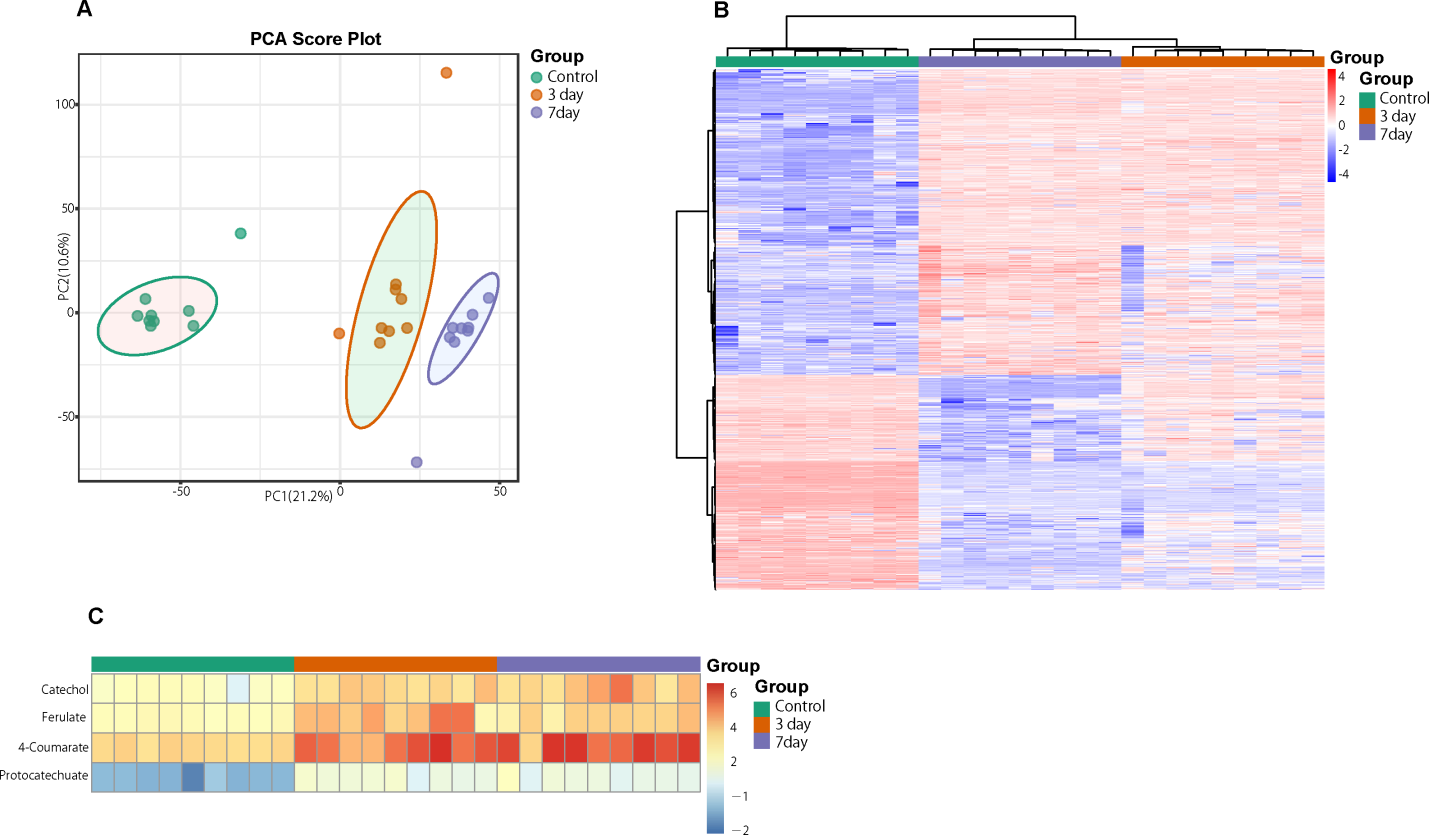
The LC-MS analysis for lignin liquid culture solution. (**A**) Scores plots of principal component analyses of identified products in lignin culture solution at 0, 3, and 7 days. (**B**) Heatmap of differential contents in lignin culture solution at 0, 3, and 7 days. (**C**) Heatmap of catechol, ferulate, 4-coumarate, and protocatechuate in lignin culture solution at 0, 3, and 7 days.

The three *Pseudomonas*-associated isolates secreted extracellular ligninolytic enzymes and produced a transparent ring after 24 h of incubation on aniline blue solid medium (Fig. 7A). However, most aniline blue was decolorized by bacteria after 48 h (Fig. 7A). The aniline blue degrading curve of *Pseudomonas putida*, *Pseudomonas* sp., and *Pseudomonas oryzihabitans* are shown in Fig. 7B. The degradation rates of *Pseudomonas putida*, *Pseudomonas* sp., and *Pseudomonas oryzihabitans* after 96 h were 90.74%, 88.97%, and 86.77%, respectively. The enzyme production curve of three *Pseudomonas*-associated isolates is shown in Fig. 8C through E. The results showed that the three *Pseudomonas*-associated isolates secreted considerable reactive Lac, LiP, and MnP. Furthermore, the three *Pseudomonas*-associated isolates showed a similar characteristic of secreting extracellular ligninolytic enzymes in the culture medium with bamboo powder as the sole carbon source. The activities of Lac, LiP, and MnP reached the maximum values after 72 h. The maximum values of Lac, LiP, and MnP were 688.25 U/L, 3065.37 U/L, and 493.22 U/L, respectively, for *Pseudomonas putida*; 653.75 U/L, 2700.54 U/L, and 439.53 U/L, respectively, for *Pseudomonas* sp.; and 459.12 U/L, 2668.54 U/L, and 404.26 U/L, respectively, for *Pseudomonas oryzihabitans*. The average Lac activity of *Pseudomonas putida*, *Pseudomonas* sp., and *Pseudomonas oryzihabitans* was 309.51, 251.58, and 209.52 U/L, respectively (Fig. 7C), while the average MnP activity was 300.08, 269.84, and 258.13 U/mL, respectively (Fig. 7E). The average LiP activity of *Pseudomonas putida*, *Pseudomonas* sp., and *Pseudomonas oryzihabitans* was 2130.5, 2083.12, and 2069.75 U/mL, respectively (Fig. 7D).

### Metabolites of degraded lignin by *Pseudomonas*-associated isolates

Compared with the control (0 day), LC-MS analysis detected new peaks in the culture medium after treatment with *Pseudomonas*-associated strain for 3 and 7 days (Fig. S4A). The PCA score plot displayed three clusters that corresponded to different cultivation time with *Pseudomonas*-associated strain. Metabolites in the culture medium after treatment with *Pseudomonas*-associated strain were significantly different compared with the control (0 day) (Fig. 8A). Distinctive clustering in the metabolite composition of the supernatant was detected between the control group (0 days) and the culture group (Fig. S4B). A total of 229 and 245 differential metabolites were detected between 0 day vs 3 days and 0 day vs 7 days, respectively, of which 201 were shared. Compared with the control (0 day), cluster analysis indicated that the 201 metabolites in the culture medium were significantly different after treatment with *Pseudomonas*-associated strains for 3 and 7 days (Fig. 8B). LC-MS analysis detected 14 of 25 substances for the products in the beta-ketoadipate and homogentisate pathway (Fig. 1). Among them, *cis*,cis-muconate, hydroxybenzaldehyde, vanillate, phenylpyruvate, 2-hydroxyphenylacetate, homogentisate, fumarylacetoacetate, and fumaric acid were only identified in the supernatant of culture medium after treatment with *Pseudomonas putida*, *Pseudomonas* sp., and *Pseudomonas oryzihabitans* (separately) for 3 and 7 days (Table 2). Lignin derivatives, such as catechol, ferulate, 4-coumarate, and protocatechuate, were detected in the control group (Table 2). Nevertheless, the relative quantification of the above derivatives was higher on the third and seventh days (Fig. 8C).

**TABLE 2:** Partial aromatic compounds identified in the control alkali lignin medium (0 day) and the alkali lignin medium degraded by *Pseudomonas*-associated strain for 3 and 7 days^*a*^

Name	Chemical formula	Control	3 d	7 d
Catechol	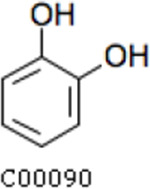	+	+	+
Ferulate	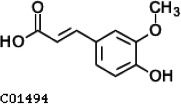	+	+	+
4-Coumarate	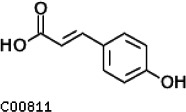	+	+	+
Protocatechuate	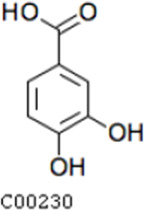	+	+	+
*cis*,cis-Muconate	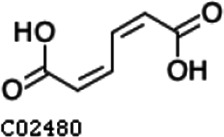	−	+	+
Hydroxybenzaldehyde	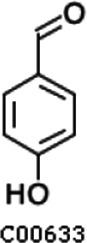	−	+	+
4-Hydroxybenzoate	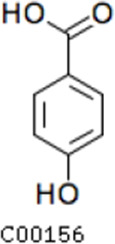	+	+	+
Vanillate	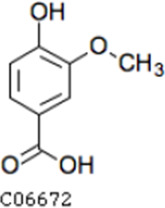	−	+	+
Vanillin	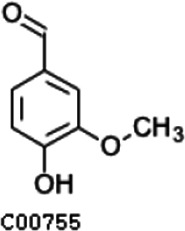	+	+	+
Phenylpyruvate	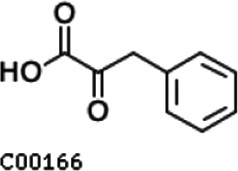	−	+	+
2-Hydroxyphenylacetate	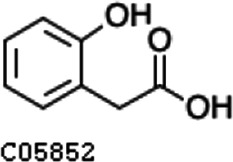	−	+	+
Homogentisate	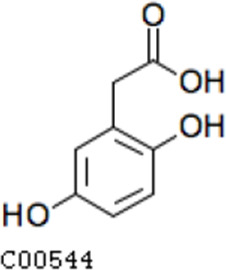	−	+	+
Fumarylacetoacetate	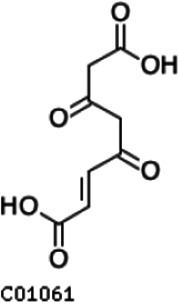	−	+	+
Fumaric acid	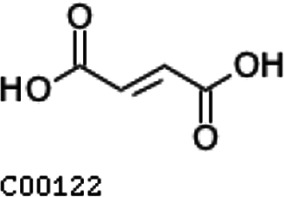	−	+	+

^*a*^ The related intermediate terminal metabolites in the metabolic pathway are shown in Fig. 1.

## DISCUSSION

Numerous studies have identified genes encoding cellulase and hemicellulose in the gut microbiota genome of giant pandas (13). However, the absence of *in vitro* experiments to validate these findings has been notable. To date, only a limited number of studies have elucidated the degradation of lignin in giant pandas, particularly the specific metabolic pathway involved in giant panda lignin degradation. The present study is the first to reveal the complete metabolic pathway involved in the degradation of lignin derivatives by the gut microbiome of giant pandas. The β-ketoadipate pathway, comprising the catechol and protocatechuate branches, is the major microbial degradation pathway for lignin-derived aromatic compounds (73, 74). Gut microbiota, such as *Rhodococcus jostii* RHA1 (75), *Brevibacillus thermoruber* (76), *Bacillus ligniniphilus* (77), and *Pseudomonas putida* KT2440 (78), degrade lignin mainly through β-KAP. In addition, the homogentisate pathway plays a key role in the degradation of lignin-derived aromatic compounds (79). Degradation pathways for 4-hydroxyphenylacetate (79), phenylalanine, and tyrosine eventually join the homogentisate pathway (80). Notably, genes involved in the β-KAP and homogentisate pathways were enriched in the gut microbiotas of wild giant pandas. The relative abundance of these genes was significantly higher in wild giant pandas than in captive giant pandas, herbivores, carnivores, and omnivores. However, the abundance of these genes in wild giant pandas was similar to those in another bamboo-eating panda (red panda). This indicates that the gut microbiome of wild giant pandas plays a key role in catalyzing cleavage of the benzene ring through the metabolic pathway involved in breakdown of central aromatic intermediates. Of note, the gut microbiotas of wild giant pandas showed significantly enriched genes encoding several enzymes that catalyze the degradation of ferulic and *p*-coumarate to protocatechuate, which subsequently join the Pbβ-KAP pathway. Ferulic and *p*-coumarate are lignin monomers (81, 82) produced during the initial degradation reaction of lignin catalyzed by laccase (83). Laccase-like multicopper oxidase gene, which plays a similar role in degrading lignin (84), was also abundant in the feces of wild giant pandas and was mainly contributed by *Pseudomonas*-associated bacteria. Therefore, most genes implicated in lignin degradation were identified in the genome of *Pseudomonas*-associated bacteria of wild giant pandas. Notably, the final product of lignin degradation (fumarate, acetyl-CoA, and succinyl-CoA) can enter the TCA cycle to produce nutritionally important intermediates. The lignin in bamboo is mainly of the HGS type [p-hydroxyphenyl (H), vanillin (G), syringaldehyde (S)], containing a considerable amount of *p*-coumarate and ferulic (85). The β-ketoadipate pathway is the major lignin-degrading pathway in bacteria isolated from erosive bamboo slips (86, 83). This explains why the gut microbiotas of wild giant pandas had significantly high expression levels of genes encoding enzymes implicated in *p*-coumarate and ferulic degradation through the β-ketoadipate pathway. Previous studies have shown that the genome of gut microbiotas of captive giant pandas lacks genes encoding enzymes involved in lignin degradation (17). The reason for these findings is that the gut microbiome composition of captive giant pandas is significantly different from that of wild giant pandas (19). For instance, the dominant gut bacteria in wild giant pandas is *Pseudomonas*, whereas the dominant gut bacteria of captive giant pandas are *Enterobacteriaceae* and *Streptococcus* (19, 20). The wild giant pandas are exclusive bamboo specialists with almost 99% of its diet being bamboo (4, 12); however, except for bamboo, captive giant pandas are fed steamed grain mixture, fruits, and animal products (87). In addition, giant pandas can eat a wider variety of bamboo in the wild. These factors may be the possible reasons for the difference in the composition and metabolism of the microflora of giant pandas in the wild and in captivity. Of course, more research is needed in the future to explain why no lignin metabolity-related pathways have been found in captive pandas. Moreover, red pandas that exclusively eat bamboo exhibit a similar phenomenon as wild and captive population (26). The results of the present study suggest that the study of the adaptive evolution of animal intestinal flora to diet should be conducted using wild populations rather than captive populations.

The genome of *Pseudomonas*-associated strains (bin 1, bin 17, bin 33, and bin 53) in the feces of giant pandas can encode all enzymes involved in the metabolism of central aromatic intermediates. *Pseudomonas* is the most efficient lignin degradation bacterium (88). The four closest neighbors of *Pseudomonas*-associated strains in the feces of giant pandas were *Pseudomonas fluorescens* PfO-1, *Pseudomonas syringae* pv. *phaseolicola* 1448A, *Pseudomonas putida* KT2440, and *Pseudomonas aeruginosa* PAO1. *Pseudomonas fluorescens* (89), *Pseudomonas putida* (36), and *Pseudomonas aeruginosa* (90) have high potential in lignin degradation. Although *Pseudomonas syringae* is generally considered a plant pathogen (91), its genome contains genes involved in the β-ketoadipate and homogentisate pathway (92). Furthermore, *in vitro* culture experiments showed that three *Pseudomonas*-associated strains isolated from the feces of wild giant pandas have a lignin degradation ability. Besides, multicopper oxidases gene was identified in *Pseudomonas syringae (93*), further confirming that *Pseudomonas*-associated bacteria in the feces of wild giant panda play a crucial role in lignin degradation. *Pseudomonas* is the most dominant bacterium in the gut of wild giant pandas (19, 20). In this study, the abundance of *Pseudomonas*-associated bins (bin 1, bin 17, bin 33, and bin 53) was higher than other bins, implying that *Pseudomonas* is the main lignin-degrading bacteria in the gut of giant pandas. Laccase-like multicopper oxidase genes in the gut of giant pandas are mainly derived from *Pseudomonas*, consistent with the findings on microbiome abundance. Fang et al. reported that multicopper oxidase in the gut of giant pandas was derived from *Pseudomonas* sp., and the enzyme showed activity for oxidative degradation of lignin (23). The three *Pseudomonas*-associated strains could also secret extracellular Lac, Lip, and MnP in the culture medium with bamboo powder as the sole carbon source. This is the first study to show that *Pseudomonas*, the most dominant bacteria genus in the gut of giant pandas, can degrade lignin.

**Fig 9:**
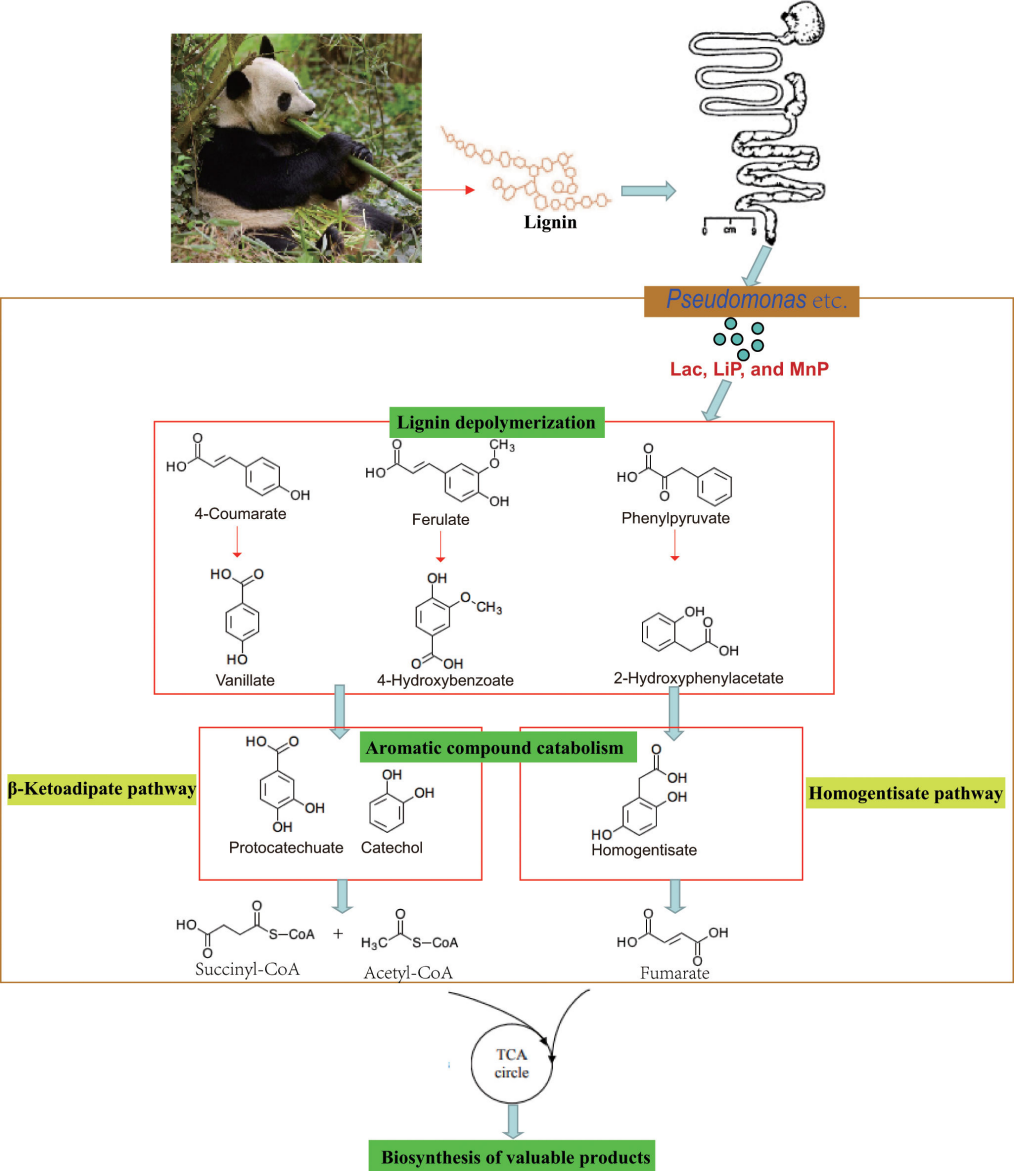
The putative model for lignin degradation by gut microbiotas of giant pandas based on the findings of this study. *Pseudomonas* genus bacteria play a central role in the model.

Based on these findings, we proposed a potential model for lignin degradation by gut microbiotas of giant pandas (Fig. 9). In this model, lignin is first depolymerized by extracellular Lac, Lip, and MnP to form lignin derivatives or monomers (*p*-coumarate, ferulate, phenylpyruvate, etc.). Lignin monomers (*p*-coumarate and ferulate, etc.) are then degraded into aromatic compounds, such as vanillate, 4-hydroxybenzoate, and 2-hydroxyphenylacetate. The aromatic compounds are further metabolized to form fumarate, acetyl-CoA, and succinyl-CoA through the β-ketoadipate pathway and homogentisate pathway. Finally, fumarate, acetyl-CoA, and succinyl-CoA enter the TCA cycle of giant pandas for the biosynthesis of metabolically important products. *Pseudomonas* is the most dominant bacteria in the gut of wild giant pandas (19, 20); thus, it plays a key role in this model.

*Pseudomonas putida* KT2440, a model strain for lignin degradation, can package enzymes implicated in lignin degradation in outer membrane vesicles (OMVs) and release them into the extracellular space to catalyze lignin degradation (36). Moreover, several *Pseudomonas* strains, including *Pseudomonas aeruginosa (94*) and *Pseudomonas syringae* (95), can release OMVs to facilitate the metabolism of lignin *in vitro*. This indicates that *Pseudomonas*-associated strains in the gut of giant pandas potentially release OMVs containing enzymes involved in lignin degradation into the extracellular space (in the gut of giant pandas) to degrade lignin. In this study, experiments of aniline blue degradation and enzyme activity determination *in vitro* confirmed that *Pseudomonas*-associated strains can release extracellular ligninolytic enzymes to degrade lignin. In addition, *Pseudomonas*-associated strains can degrade lignin into some important raw materials, such as fumarate, acetyl-CoA, and succinyl-CoA, for the TCA *in vitro*. Notably, LC-MS did not detect acetyl-CoA and succinyl-CoA in the culture solution, indicating low content or other detection methods are needed. However, these results indicate that the gut of giant panda may assimilate the final products (fumarate, acetyl-CoA, and succinyl-CoA) of lignin degradation in the extracellular space into the TCA cycle to obtain nutritionally important compounds. This hypothesis effectively explains how giant pandas potentially obtain nutrition from a low-nutrition bamboo diet. However, additional studies are needed to verify if the intestinal tract of giant pandas can absorb lignin degradation products for biosynthesis metabolism. In summary, this study provides new insights into how giant pandas obtain nutrition from bamboo.

### Conclusion

The gut microbiome of wild giant pandas exhibits high expression levels of genes implicated in lignin derivative degradation pathways. These pathways include catechol branch of beta-ketoadipate pathway, protocatechuate branch of beta-ketoadipate pathway, homogentisate pathway of aromatic compound degradation, and pathways for degradation of lignin monomers (*p*-coumarate and ferulate) into the important raw materials for TCA via beta-ketoadipate pathway, such as acetyl-CoA and succinyl-CoA. All these pathways can be found in the genome of the most dominant bacteria genus, *Pseudomonas*-associated strains. Furthermore, results showed that *Pseudomonas*-associated strains isolated from the feces of pandas can degrade extracellular lignin *in vitro*. In general, the predominant bacteria in giant pandas, particularly *Pseudomonas*, may play a crucial role as important lignin-degrading agents. Their presence may have been instrumental in facilitating the adaptation of giant pandas to a bamboo-centric diet.

## Data Availability

The raw data of metagenome sequences in this study have been deposited into Sequence Read Archive (SRA) in NCBI with the accession BioProject number PRJNA356809.

## SUPPLEMENTAL MATERIAL

Fig. S1 spectrum.03819-23-s0001.pdf PCoA of predicted metagenomic function in the gut of bamboo-eating pandas, terrestrial mammals, and wood-feeding insect when considering all KEGG genes.

Fig. S2 spectrum.03819-23-s0002.pdf Stacked bar plots illustrate the mean relative abundance of OTUs at genus level in the fecal microbiomes of wild and captive giant pandas.

Fig. S3 spectrum.03819-23-s0003.pdf Functional annotation of genome of Pseudomonas fluorescens PfO-1, Pseudomonas syringae pv. phaseolicola 1448A, Pseudomonas putida KT2440, and Pseudomonas aeruginosa PAO1.

Fig. S4 spectrum.03819-23-s0004.pdf Metabolome analysis of Pseudomonas in lignin culture medium.

Table S1 spectrum.03819-23-s0005.pdf Detailed information of sampling in this study.

Table S2 spectrum.03819-23-s0006.pdf Published raw metagenome sequences used in this study.

Table S3 spectrum.03819-23-s0007.pdf Statistics on the taxonomic assignment of enzymes involved in degrading lignin derivatives from wild giant pandas.

Table S4 spectrum.03819-23-s0008.pdf Information of individual draft genomes recovered from the gut metagenomic data of wild giant pandas in this study.

Table S5 spectrum.03819-23-s0009.pdf Genes involved in lignin degradation identified in the bins retrieved from wild giant pandas.

Table S6 spectrum.03819-23-s0010.pdf Taxonomic assignment of laccase-like multicopper oxidase genes in the feces of wild giant pandas.

ASM does not own the copyrights to Supplemental Material that may be linked to, or accessed through, an article. The authors have granted ASM a non-exclusive, world-wide license to publish the Supplemental Material files. Please contact the corresponding author directly for reuse.
