# Supplementary material for: Pseudomonas-associated bacteria play a key role in obtaining nutrition from bamboo for the giant panda (Ailuropoda melanoleuca)
Source: Microbiol Spectr. 2024 Feb 2;12(3):e03819-23. doi: 10.1128/spectrum.03819-23 (PMC10913395; doi:10.1128/spectrum.03819-23)

Fig. S1 Principle Coordinate Analysis (PCoA) of predicted metagenomic function in the gut of bamboo-eating pandas, terrestrial mammals and wood-feeding insect when considering all KEGG genes.

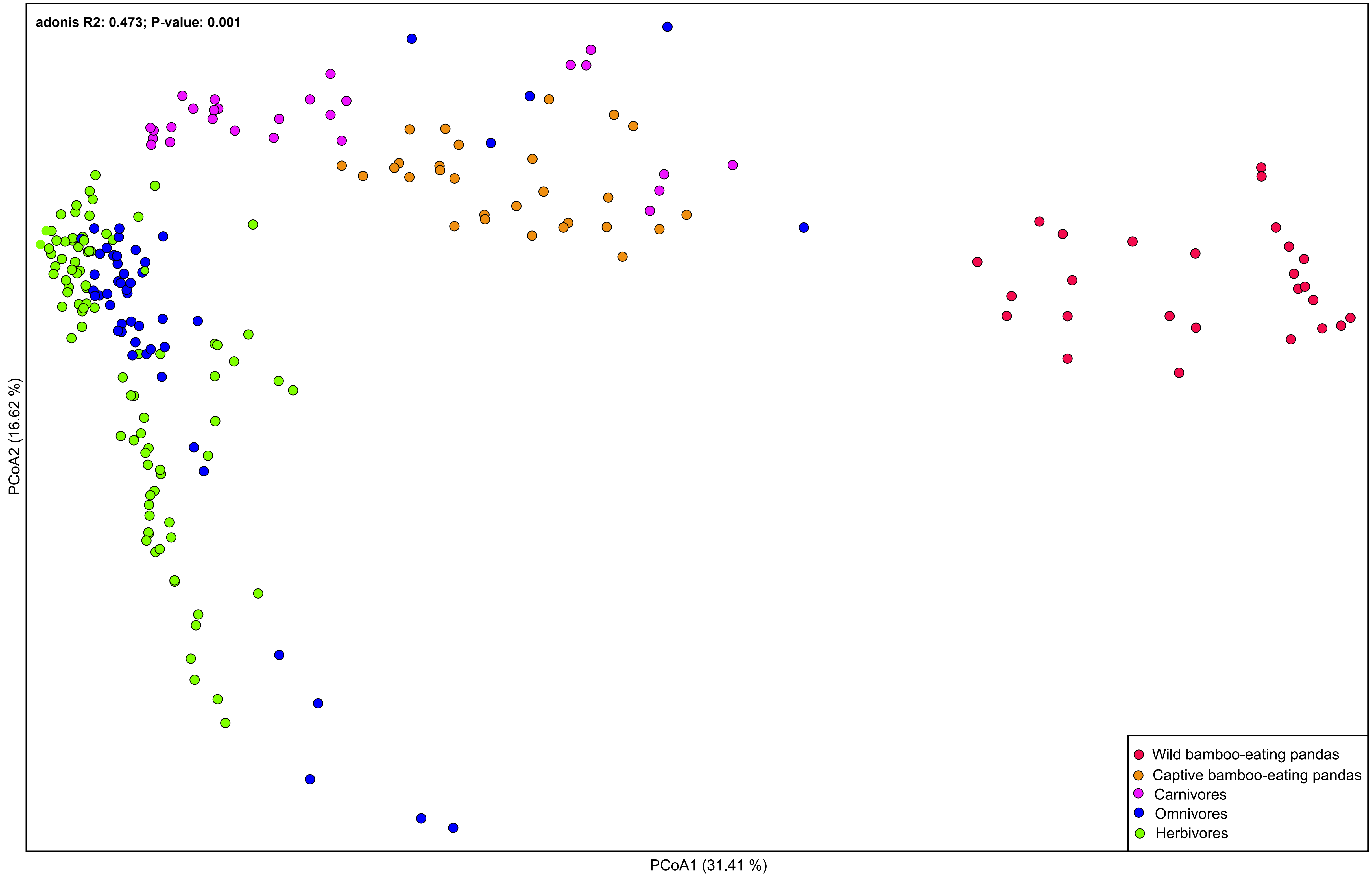

Supplement: Fig. S1 — PCoA of predicted metagenomic function in the gut of bamboo-eating pandas, terrestrial mammals, and wood-feeding insect when considering all KEGG genes. [file spectrum.03819-23-s0001.pdf]
