# Supplementary material for: Pseudomonas-associated bacteria play a key role in obtaining nutrition from bamboo for the giant panda (Ailuropoda melanoleuca)
Source: Microbiol Spectr. 2024 Feb 2;12(3):e03819-23. doi: 10.1128/spectrum.03819-23 (PMC10913395; doi:10.1128/spectrum.03819-23)

Fig. S2 Stacked bar plots illustrate the mean relative abundance of OTUs at genus (top 20 OTUs) level in the fecal microbiomes of wild (A) and Captive (B) giant pandas.

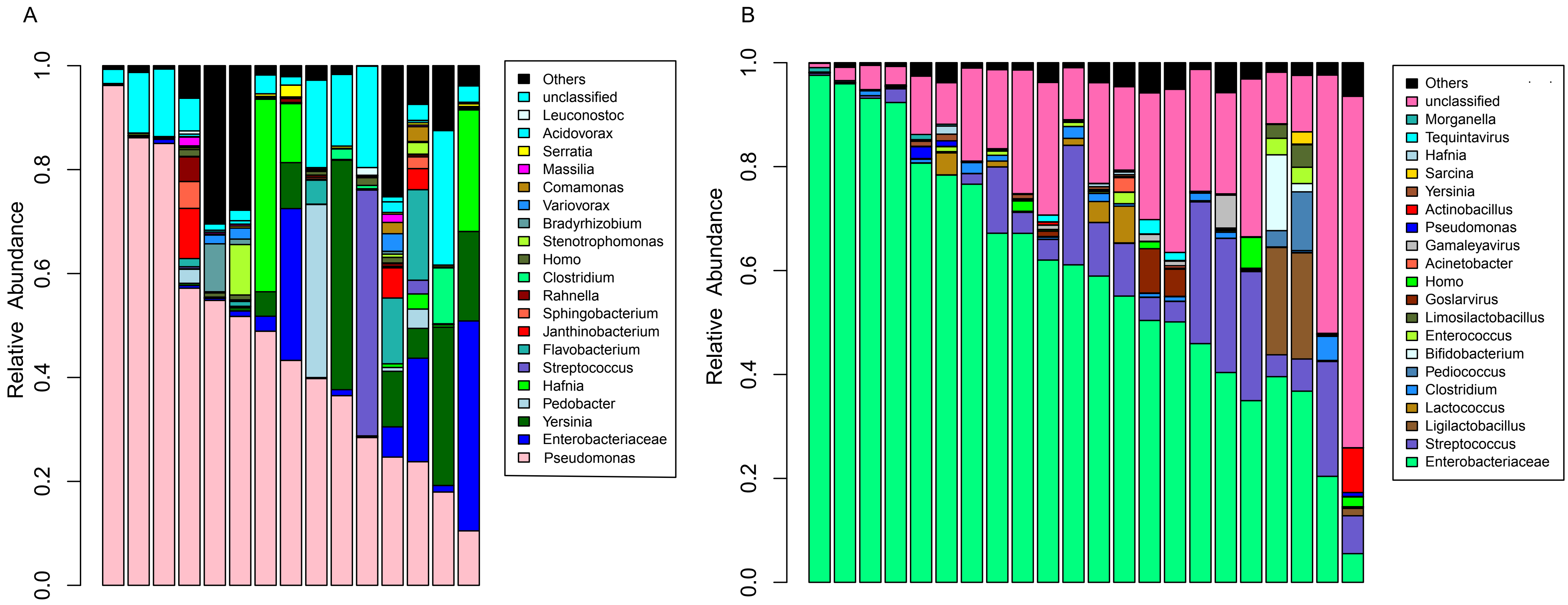

Supplement: Fig. S2 — Stacked bar plots illustrate the mean relative abundance of OTUs at genus level in the fecal microbiomes of wild and captive giant pandas. [file spectrum.03819-23-s0002.pdf]
