# Supplementary material for: Pseudomonas-associated bacteria play a key role in obtaining nutrition from bamboo for the giant panda (Ailuropoda melanoleuca)
Source: Microbiol Spectr. 2024 Feb 2;12(3):e03819-23. doi: 10.1128/spectrum.03819-23 (PMC10913395; doi:10.1128/spectrum.03819-23)

Fig. S3 The functional annotation of genome of Pseudomonas fluorescens PfO-1 (A), Pseudomonas syringae pv. phaseolicola 1448A (B), Pseudomonas putida KT2440 (C) and Pseudomonas aeruginosa PAO1 (D).

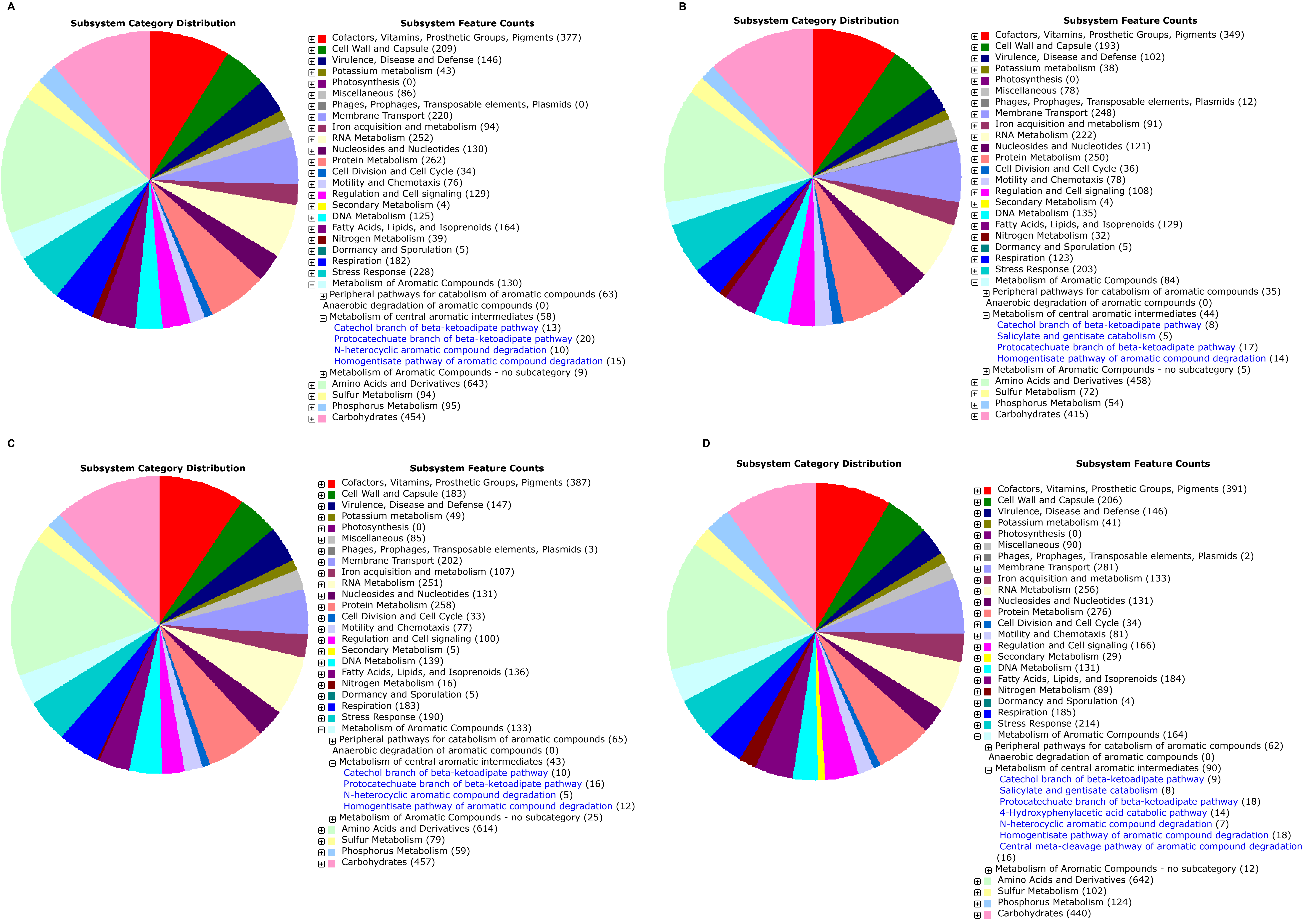

Supplement: Fig. S3 — Functional annotation of genome of Pseudomonas fluorescens PfO-1, Pseudomonas syringae pv. phaseolicola 1448A, Pseudomonas putida KT2440, and Pseudomonas aeruginosa PAO1. [file spectrum.03819-23-s0003.pdf]
