# Supplementary material for: Pseudomonas-associated bacteria play a key role in obtaining nutrition from bamboo for the giant panda (Ailuropoda melanoleuca)
Source: Microbiol Spectr. 2024 Feb 2;12(3):e03819-23. doi: 10.1128/spectrum.03819-23 (PMC10913395; doi:10.1128/spectrum.03819-23)

Fig. S4 Metabolome analysis of Pseudomonas in lignin culture medium. A, Base Peak Chromatogram for lignin culture solution treated with Pseudomonas-associated strain at 0, 3, and 7day. B, Heatmap of identified contents in lignin culture solution at 0, 3, and 7day.

A

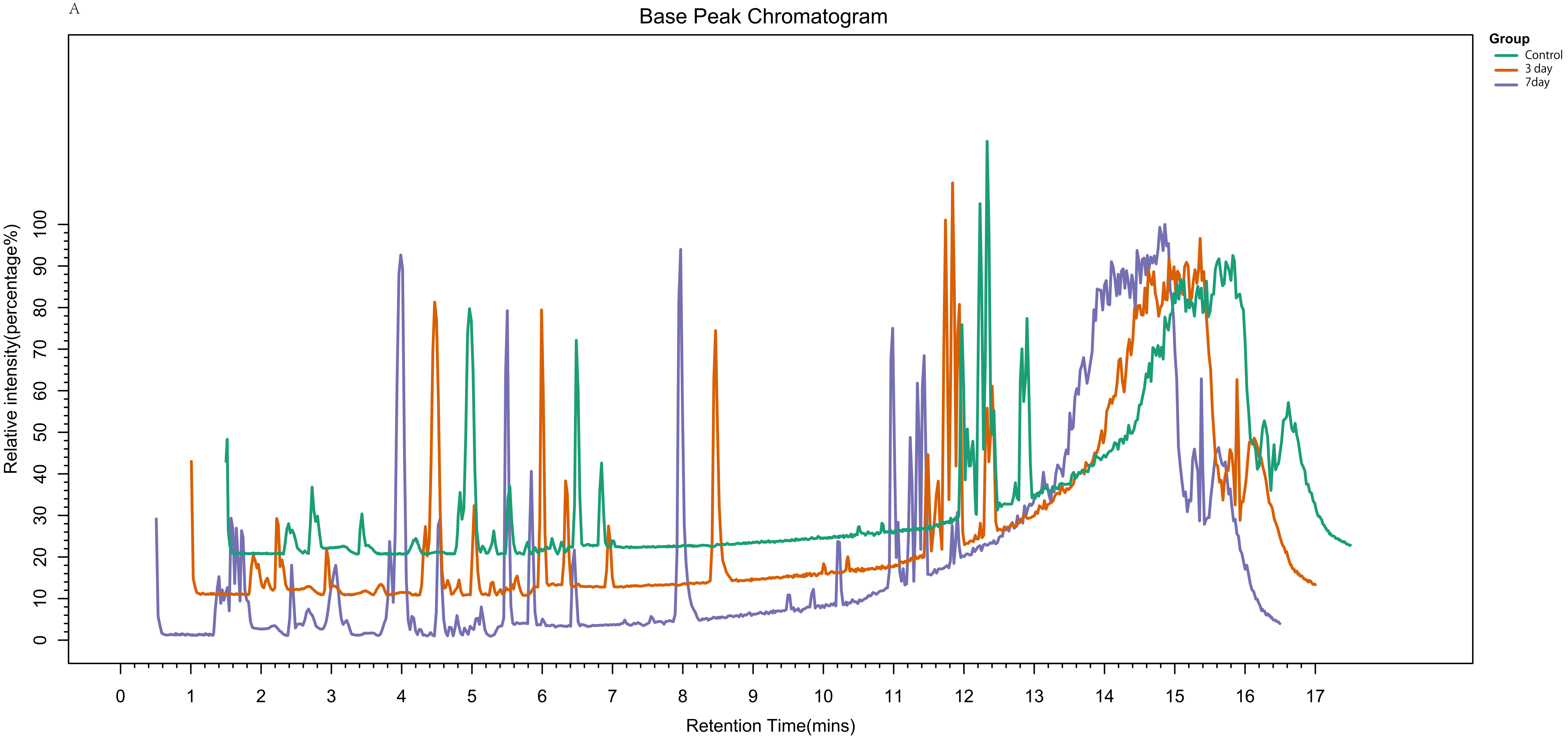

B

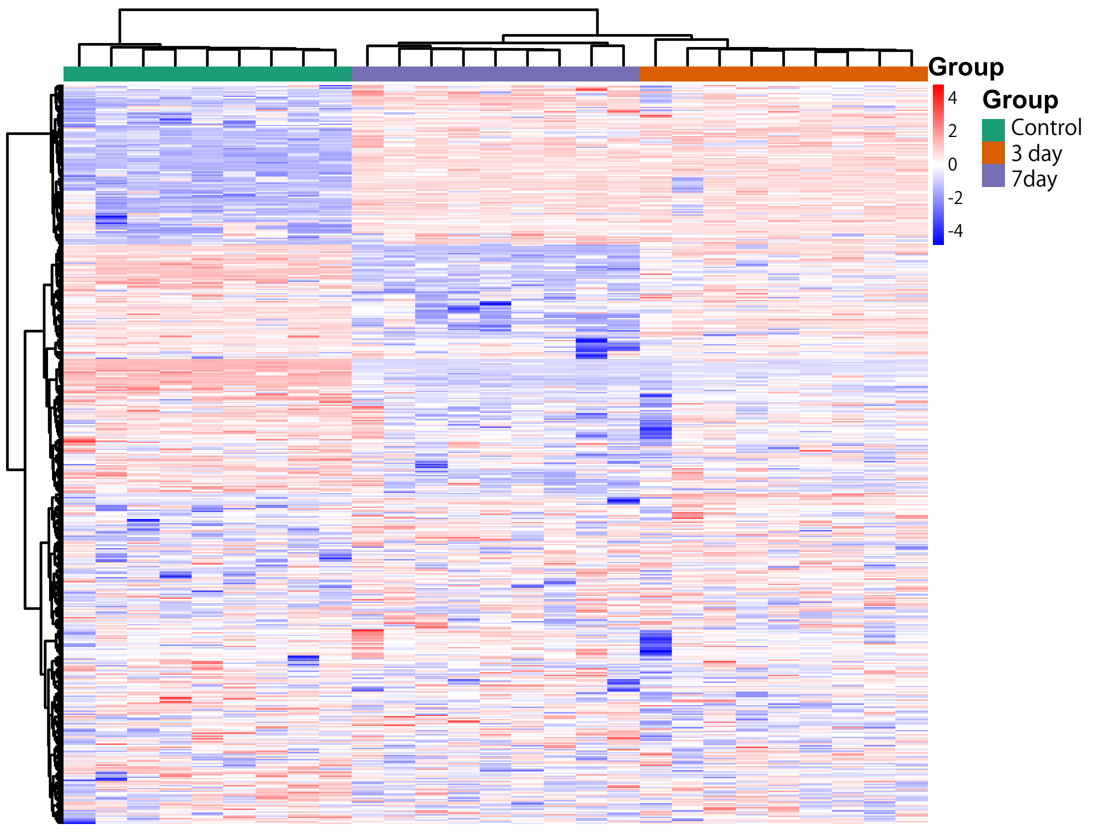

Supplement: Fig. S4 — Metabolome analysis of Pseudomonas in lignin culture medium. [file spectrum.03819-23-s0004.pdf]
