# Supplementary material for: Pseudomonas-associated bacteria play a key role in obtaining nutrition from bamboo for the giant panda (Ailuropoda melanoleuca)
Source: Microbiol Spectr. 2024 Feb 2;12(3):e03819-23. doi: 10.1128/spectrum.03819-23 (PMC10913395; doi:10.1128/spectrum.03819-23)
Supplement: Table S1 — Detailed information of sampling in this study. [file spectrum.03819-23-s0005.pdf]

Table S1. Detailed information of sampling in this study.

Sample ID	Host scientific name	Host common name	Collection date	Location	Coordinates	Source
WP1	Ailuropoda melanoleuca	Giant panda	Nov 2013	Baoxing, Sichuan, China	102.8983,30.4575	Fengtongzhai National Nature Reserve
WP3	Ailuropoda melanoleuca	Giant panda	Nov 2013	Baoxing, Sichuan, China	102.8724,30.3361	Fengtongzhai National Nature Reserve
WP4	Ailuropoda melanoleuca	Giant panda	Nov 2013	Baoxing, Sichuan, China	102.8438,30.6936	Fengtongzhai National Nature Reserve
WP5	Ailuropoda melanoleuca	Giant panda	Nov 2013	Baoxing, Sichuan, China	102.9082,30.3812	Fengtongzhai National Nature Reserve
WP10	Ailuropoda melanoleuca	Giant panda	Nov 2013	Baoxing, Sichuan, China	102.5465,30.3656	Fengtongzhai National Nature Reserve
WP12	Ailuropoda melanoleuca	Giant panda	Nov 2013	Baoxing, Sichuan, China	102.9610,30.6001	Fengtongzhai National Nature Reserve
WP14	Ailuropoda melanoleuca	Giant panda	Nov 2013	Baoxing, Sichuan, China	102.8842,30.6298	Fengtongzhai National Nature Reserve
WR3	Ailurus fulgens	Red panda	Dec 2014	Baoxing, Sichuan, China	102.8927,30.6194	Fengtongzhai National Nature Reserve
WR4	Ailurus fulgens	Red panda	Dec 2014	Baoxing, Sichuan, China	102.5328,30.3645	Fengtongzhai National Nature Reserve
WR8	Ailurus fulgens	Red panda	Dec 2014	Baoxing, Sichuan, China	102.8097,30.6158	Fengtongzhai National Nature Reserve
WR10	Ailurus fulgens	Red panda	Dec 2014	Baoxing, Sichuan, China	102.5331,30.3653	Fengtongzhai National Nature Reserve
WR15	Ailurus fulgens	Red panda	Dec 2014	Baoxing, Sichuan, China	102.5341,30.3653	Fengtongzhai National Nature Reserve
FY1.27	Ailuropoda melanoleuca	Giant panda	Jan 2015	Yaan, Sichuan, China	102.8983,30.37753	China Conservation and Research Center for the Giant Panda
DL1	Ailuropoda melanoleuca	Giant panda	Jan 2015	Dujiangyan,Sichuan, China	103.5777, 30.85956	China Conservation and Research Center for the Giant Panda
WG	Ailuropoda melanoleuca	Giant panda	Jan 2015	Yaan, Sichuan, China	102.8983,30.37753	China Conservation and Research Center for the Giant Panda
GZ1.9.2	Ailuropoda melanoleuca	Giant panda	Jan 2015	Dujiangyan,Sichuan, China	103.5777, 30.85956	China Conservation and Research Center for the Giant Panda
ZM2.1.27	Ailuropoda melanoleuca	Giant panda	Jan 2015	Yaan, Sichuan, China	102.8983,30.37753	China Conservation and Research Center for the Giant Panda
HH	Ailuropoda melanoleuca	Giant panda	Jan 2015	Dujiangyan,Sichuan, China	103.5777, 30.85956	China Conservation and Research Center for the Giant Panda
JX	Ailuropoda melanoleuca	Giant panda	Jan 2015	Yaan, Sichuan, China	102.8983,30.37753	China Conservation and Research Center for the Giant Panda
CR2	Ailurus fulgens	Giant panda	Jan 2015	Yaan, Sichuan, China	102.8983,30.37753	Bifengxia Ecological Zoo
CR6	Ailurus fulgens	Giant panda	Jan 2015	Yaan, Sichuan, China	102.8983,30.37753	Bifengxia Ecological Zoo
ZR1	Ailurus fulgens	Giant panda	Jan 2015	Yaan, Sichuan, China	102.8983,30.37753	Bifengxia Ecological Zoo
ZR7	Ailurus fulgens	Giant panda	Jan 2015	Yaan, Sichuan, China	102.8983,30.37753	Bifengxia Ecological Zoo
ZR8	Ailurus fulgens	Giant panda	Jan 2015	Yaan, Sichuan, China	102.8983,30.37753	Bifengxia Ecological Zoo
