# Supplementary material for: Pseudomonas-associated bacteria play a key role in obtaining nutrition from bamboo for the giant panda (Ailuropoda melanoleuca)
Source: Microbiol Spectr. 2024 Feb 2;12(3):e03819-23. doi: 10.1128/spectrum.03819-23 (PMC10913395; doi:10.1128/spectrum.03819-23)
Supplement: Table S2 — Published raw metagenome sequences used in this study. [file spectrum.03819-23-s0006.pdf]

Table S2. The published raw metagenome sequences used in this study.

Sample ID	Host scientific name	Wild/Captive	Diet	Accession number	Sequencing platform	Source
SRR7340327	Ailuropoda melanoleuca	Wild	Bamboo	PRJNA475184	Illumina Hiseq	Zhu et al.(2018)
SRR7340328	Ailuropoda melanoleuca	Wild	Bamboo	PRJNA475184	Illumina Hiseq	Zhu et al.(2018)
SRR7340341	Ailuropoda melanoleuca	Wild	Bamboo	PRJNA475184	Illumina Hiseq	Zhu et al.(2018)
SRR7340342	Ailuropoda melanoleuca	Wild	Bamboo	PRJNA475184	Illumina Hiseq	Zhu et al.(2018)
SRR7340343	Ailuropoda melanoleuca	Wild	Bamboo	PRJNA475184	Illumina Hiseq	Zhu et al.(2018)
SRR7340344	Ailuropoda melanoleuca	Wild	Bamboo	PRJNA475184	Illumina Hiseq	Zhu et al.(2018)
SRR7340346	Ailuropoda melanoleuca	Wild	Bamboo	PRJNA475184	Illumina Hiseq	Zhu et al.(2018)
SRR7340347	Ailuropoda melanoleuca	Wild	Bamboo	PRJNA475184	Illumina Hiseq	Zhu et al.(2018)
SRR7340334	Ailurus fulgens	Wild	Bamboo	PRJNA475184	Illumina Hiseq	Zhu et al.(2018)
SRR7340335	Ailurus fulgens	Wild	Bamboo	PRJNA475184	Illumina Hiseq	Zhu et al.(2018)
SRR7340337	Ailurus fulgens	Wild	Bamboo	PRJNA475184	Illumina Hiseq	Zhu et al.(2018)
SRR7340338	Ailurus fulgens	Wild	Bamboo	PRJNA475184	Illumina Hiseq	Zhu et al.(2018)
SRR7340339	Ailurus fulgens	Wild	Bamboo	PRJNA475184	Illumina Hiseq	Zhu et al.(2018)
SRR7340340	Ailurus fulgens	Wild	Bamboo	PRJNA475184	Illumina Hiseq	Zhu et al.(2018)
SRR8890819	Diceros bicornis	Wild	Herbivorous	PRJNA532626	Illumina Hiseq	Gibson et al. (2019)
SRR8890820	Diceros bicornis	Wild	Herbivorous	PRJNA532626	Illumina Hiseq	Gibson et al. (2019)
SRR8890821	Diceros bicornis	Wild	Herbivorous	PRJNA532626	Illumina Hiseq	Gibson et al. (2019)
SRR8890822	Diceros bicornis	Wild	Herbivorous	PRJNA532626	Illumina Hiseq	Gibson et al. (2019)
SRR8890823	Diceros bicornis	Wild	Herbivorous	PRJNA532626	Illumina Hiseq	Gibson et al. (2019)
SRR8890824	Diceros bicornis	Wild	Herbivorous	PRJNA532626	Illumina Hiseq	Gibson et al. (2019)
SRR8890825	Diceros bicornis	Wild	Herbivorous	PRJNA532626	Illumina Hiseq	Gibson et al. (2019)
SRR8890826	Diceros bicornis	Wild	Herbivorous	PRJNA532626	Illumina Hiseq	Gibson et al. (2019)
SRR8890827	Diceros bicornis	Wild	Herbivorous	PRJNA532626	Illumina Hiseq	Gibson et al. (2019)
SRR8890828	Diceros bicornis	Wild	Herbivorous	PRJNA532626	Illumina Hiseq	Gibson et al. (2019)
SRR8890829	Diceros bicornis	Wild	Herbivorous	PRJNA532626	Illumina Hiseq	Gibson et al. (2019)
SRR8890830	Diceros bicornis	Wild	Herbivorous	PRJNA532626	Illumina Hiseq	Gibson et al. (2019)
SRR8890831	Diceros bicornis	Wild	Herbivorous	PRJNA532626	Illumina Hiseq	Gibson et al. (2019)
SRR8890832	Diceros bicornis	Wild	Herbivorous	PRJNA532626	Illumina Hiseq	Gibson et al. (2019)
SRR8890833	Diceros bicornis	Wild	Herbivorous	PRJNA532626	Illumina Hiseq	Gibson et al. (2019)
SRR8890834	Diceros bicornis	Wild	Herbivorous	PRJNA532626	Illumina Hiseq	Gibson et al. (2019)
SRR8890835	Diceros bicornis	Wild	Herbivorous	PRJNA532626	Illumina Hiseq	Gibson et al. (2019)
SRR7340300	Elaphurus davidianus	Wild	Herbivorous	PRJNA475184	Illumina Hiseq	Zhu et al.(2018)
SRR7340301	Elaphurus davidianus	Wild	Herbivorous	PRJNA475184	Illumina Hiseq	Zhu et al.(2018)
SRR7340302	Elaphurus davidianus	Wild	Herbivorous	PRJNA475184	Illumina Hiseq	Zhu et al.(2018)
SRR7340303	Elaphurus davidianus	Wild	Herbivorous	PRJNA475184	Illumina Hiseq	Zhu et al.(2018)
SRR7340304	Elaphurus davidianus	Wild	Herbivorous	PRJNA475184	Illumina Hiseq	Zhu et al.(2018)
SRR7340305	Elaphurus davidianus	Wild	Herbivorous	PRJNA475184	Illumina Hiseq	Zhu et al.(2018)
SRR7340306	Elaphurus davidianus	Wild	Herbivorous	PRJNA475184	Illumina Hiseq	Zhu et al.(2018)
SRR7340307	Elaphurus davidianus	Wild	Herbivorous	PRJNA475184	Illumina Hiseq	Zhu et al.(2018)

SRR7340308	Elaphurus davidianus	Wild	Herbivorous	PRJNA475184	Illumina Hiseq	Zhu et al.(2018)
SRR7340309	Elaphurus davidianus	Wild	Herbivorous	PRJNA475184	Illumina Hiseq	Zhu et al.(2018)
SRR7340310	Elaphurus davidianus	Wild	Herbivorous	PRJNA475184	Illumina Hiseq	Zhu et al.(2018)
SRR7340311	Elaphurus davidianus	Wild	Herbivorous	PRJNA475184	Illumina Hiseq	Zhu et al.(2018)
SRR7340312	Elaphurus davidianus	Wild	Herbivorous	PRJNA475184	Illumina Hiseq	Zhu et al.(2018)
SRR7340313	Elaphurus davidianus	Wild	Herbivorous	PRJNA475184	Illumina Hiseq	Zhu et al.(2018)
SRR7340316	Elaphurus davidianus	Wild	Herbivorous	PRJNA475184	Illumina Hiseq	Zhu et al.(2018)
SRR7340317	Elaphurus davidianus	Wild	Herbivorous	PRJNA475184	Illumina Hiseq	Zhu et al.(2018)
SRR7340318	Elaphurus davidianus	Wild	Herbivorous	PRJNA475184	Illumina Hiseq	Zhu et al.(2018)
SRR7340319	Elaphurus davidianus	Wild	Herbivorous	PRJNA475184	Illumina Hiseq	Zhu et al.(2018)
SRR7340320	Elaphurus davidianus	Wild	Herbivorous	PRJNA475184	Illumina Hiseq	Zhu et al.(2018)
SRR7340321	Elaphurus davidianus	Wild	Herbivorous	PRJNA475184	Illumina Hiseq	Zhu et al.(2018)
SRR7340322	Elaphurus davidianus	Wild	Herbivorous	PRJNA475184	Illumina Hiseq	Zhu et al.(2018)
SRR7340323	Elaphurus davidianus	Wild	Herbivorous	PRJNA475184	Illumina Hiseq	Zhu et al.(2018)
SRR7340324	Elaphurus davidianus	Wild	Herbivorous	PRJNA475184	Illumina Hiseq	Zhu et al.(2018)
SRR7340325	Elaphurus davidianus	Wild	Herbivorous	PRJNA475184	Illumina Hiseq	Zhu et al.(2018)
SRR7340336	Elaphurus davidianus	Wild	Herbivorous	PRJNA475184	Illumina Hiseq	Zhu et al.(2018)
SRR7340345	Elaphurus davidianus	Wild	Herbivorous	PRJNA475184	Illumina Hiseq	Zhu et al.(2018)
SRR7340348	Elaphurus davidianus	Wild	Herbivorous	PRJNA475184	Illumina Hiseq	Zhu et al.(2018)
SRR7340349	Elaphurus davidianus	Wild	Herbivorous	PRJNA475184	Illumina Hiseq	Zhu et al.(2018)
SRR7340350	Elaphurus davidianus	Wild	Herbivorous	PRJNA475184	Illumina Hiseq	Zhu et al.(2018)
SRR7340351	Elaphurus davidianus	Wild	Herbivorous	PRJNA475184	Illumina Hiseq	Zhu et al.(2018)
SRR9995036	Pantholops hodgsoni	Wild	Herbivorous	SRR9995036	Illumina HiSeq	Qin et al.(2022)
SRR9995037	Pantholops hodgsoni	Wild	Herbivorous	SRR9995037	Illumina HiSeq	Qin et al.(2022)
SRR9995038	Pantholops hodgsoni	Wild	Herbivorous	SRR9995038	Illumina HiSeq	Qin et al.(2022)
SRR9995039	Pantholops hodgsoni	Wild	Herbivorous	SRR9995039	Illumina HiSeq	Qin et al.(2022)
CRR281845	Manis pentadactyla	Wild	Carnivorous	CRR281845	Illumina HiSeq	Liu et al.(2021)
CRR281846	Manis pentadactyla	Wild	Carnivorous	CRR281846	Illumina HiSeq	Liu et al.(2021)
CRR281847	Manis pentadactyla	Wild	Carnivorous	CRR281847	Illumina HiSeq	Liu et al.(2021)
CRR281848	Manis pentadactyla	Wild	Carnivorous	CRR281848	Illumina HiSeq	Liu et al.(2021)
CRR281849	Manis pentadactyla	Wild	Carnivorous	CRR281849	Illumina HiSeq	Liu et al.(2021)
CRR281829	Manis javanica	Wild	Carnivorous	CRR281829	Illumina HiSeq	Liu et al.(2021)
CRR281830	Manis javanica	Wild	Carnivorous	CRR281830	Illumina HiSeq	Liu et al.(2021)
CRR281831	Manis javanica	Wild	Carnivorous	CRR281831	Illumina HiSeq	Liu et al.(2021)
CRR281832	Manis javanica	Wild	Carnivorous	CRR281832	Illumina HiSeq	Liu et al.(2021)
CRR281833	Manis javanica	Wild	Carnivorous	CRR281833	Illumina HiSeq	Liu et al.(2021)
SRR6155883	Panthera tigris	Wild	Carnivorous	SRR6155883	Illumina HiSeq	Ning et al.(2020)
SRR6155884	Panthera tigris	Wild	Carnivorous	SRR6155884	Illumina HiSeq	Ning et al.(2020)
SRR6155885	Panthera tigris	Wild	Carnivorous	SRR6155885	Illumina HiSeq	Ning et al.(2020)
SRR6256454	Panthera tigris	Wild	Carnivorous	SRR6256454	Illumina HiSeq	Ning et al.(2020)
SRR6256455	Panthera tigris	Wild	Carnivorous	SRR6256455	Illumina HiSeq	Ning et al.(2020)
SRR6256456	Panthera tigris	Wild	Carnivorous	SRR6256456	Illumina HiSeq	Ning et al.(2020)
11641712540006	Gorilla beringei	Wild	Herbivorous	11641712540006	Illumina HiSeq	Levin et al.(2021)
11641712540019	Gorilla beringei	Wild	Herbivorous	11641712540019	Illumina HiSeq	Levin et al.(2021)

11641712540042	Propithecus verreauxi	Wild	Herbivorous	11641712540042	Illumina HiSeq	Levin et al.(2021)
11641712540056	Kobus kob	Wild	Herbivorous	11641712540056	Illumina HiSeq	Levin et al.(2021)
11641712540057	Chlorocebus pygerythrus	Wild	Herbivorous	11641712540057	Illumina HiSeq	Levin et al.(2021)
11641712540059	Phacochoerus africanus	Wild	Herbivorous	11641712540059	Illumina HiSeq	Levin et al.(2021)
11641712540061	Colobus guereza	Wild	Herbivorous	11641712540061	Illumina HiSeq	Levin et al.(2021)
11641712540066	Chlorocebus pygerythrus	Wild	Herbivorous	11641712540066	Illumina HiSeq	Levin et al.(2021)
11641712540068	Equus burchellii	Wild	Herbivorous	11641712540068	Illumina HiSeq	Levin et al.(2021)
11641712540070	Pan troglodytes	Wild	Herbivorous	11641712540070	Illumina HiSeq	Levin et al.(2021)
11641712540071	Colobus guereza	Wild	Herbivorous	11641712540071	Illumina HiSeq	Levin et al.(2021)
11641712540088	Propithecus verreauxi	Wild	Herbivorous	11641712540088	Illumina HiSeq	Levin et al.(2021)
11641712540142	Propithecus verreauxi	Wild	Herbivorous	11641712540142	Illumina HiSeq	Levin et al.(2021)
11641712540144	Prolemur simus	Wild	Herbivorous	11641712540144	Illumina HiSeq	Levin et al.(2021)
11641712540150	Cryptoprocta ferox	Wild	Herbivorous	11641712540150	Illumina HiSeq	Levin et al.(2021)
11641712540159	Eulemur rufifrons	Wild	Herbivorous	11641712540159	Illumina HiSeq	Levin et al.(2021)
11641712540163	Eulemur rufifrons	Wild	Herbivorous	11641712540163	Illumina HiSeq	Levin et al.(2021)
22001708574196	Erinaceus europaeus	Wild	Herbivorous	22001708574196	Illumina HiSeq	Levin et al.(2021)
22001801631157	Hapalemur aureus	Wild	Herbivorous	22001801631157	Illumina HiSeq	Levin et al.(2021)
22001801631161	Eulemur rubriventer	Wild	Herbivorous	22001801631161	Illumina HiSeq	Levin et al.(2021)
22001801631164	Propithecus edwardsi	Wild	Herbivorous	22001801631164	Illumina HiSeq	Levin et al.(2021)
22001801631167	Gorilla beringei	Wild	Herbivorous	22001801631167	Illumina HiSeq	Levin et al.(2021)
22001801631168	Hapalemur aureus	Wild	Herbivorous	22001801631168	Illumina HiSeq	Levin et al.(2021)
22001801631170	Propithecus edwardsi	Wild	Herbivorous	22001801631170	Illumina HiSeq	Levin et al.(2021)
22001801631177	Eulemur rubriventer	Wild	Herbivorous	22001801631177	Illumina HiSeq	Levin et al.(2021)
22001801631947	Ourebia ourebi	Wild	Herbivorous	22001801631947	Illumina HiSeq	Levin et al.(2021)
22001801631948	Ceratotherium simum	Wild	Herbivorous	22001801631948	Illumina HiSeq	Levin et al.(2021)
22001801631949	Alcelaphus buselaphus	Wild	Herbivorous	22001801631949	Illumina HiSeq	Levin et al.(2021)
22001801631963	Hippopotamus amphibius	Wild	Herbivorous	22001801631963	Illumina HiSeq	Levin et al.(2021)
22001801631964	Ourebia ourebi	Wild	Herbivorous	22001801631964	Illumina HiSeq	Levin et al.(2021)
22001801631966	Kobus ellipsiprymnus	Wild	Herbivorous	22001801631966	Illumina HiSeq	Levin et al.(2021)
22001801631967	Syncerus caffer	Wild	Herbivorous	22001801631967	Illumina HiSeq	Levin et al.(2021)
22001801631969	Ourebia ourebi	Wild	Herbivorous	22001801631969	Illumina HiSeq	Levin et al.(2021)
22001801631973	Alcelaphus buselaphus	Wild	Herbivorous	22001801631973	Illumina HiSeq	Levin et al.(2021)
22001801631977	Ceratotherium simum	Wild	Herbivorous	22001801631977	Illumina HiSeq	Levin et al.(2021)
22001801631980	Ceratotherium simum	Wild	Herbivorous	22001801631980	Illumina HiSeq	Levin et al.(2021)
22001801631982	Giraffa camelopardalis	Wild	Herbivorous	22001801631982	Illumina HiSeq	Levin et al.(2021)
22001801631993	Damaliscus lunatus	Wild	Herbivorous	22001801631993	Illumina HiSeq	Levin et al.(2021)
22001801632001	Eulemur rubriventer	Wild	Herbivorous	22001801632001	Illumina HiSeq	Levin et al.(2021)
22001801632002	Chlorocebus pygerythrus	Wild	Herbivorous	22001801632002	Illumina HiSeq	Levin et al.(2021)
22001801632003	Giraffa camelopardalis	Wild	Herbivorous	22001801632003	Illumina HiSeq	Levin et al.(2021)
22001801632011	Equus burchellii	Wild	Herbivorous	22001801632011	Illumina HiSeq	Levin et al.(2021)
22001801632013	Loxodonta africana	Wild	Herbivorous	22001801632013	Illumina HiSeq	Levin et al.(2021)
22001801632016	Microcebus murinus	Wild	Herbivorous	22001801632016	Illumina HiSeq	Levin et al.(2021)
22001801632017	Kobus ellipsiprymnus	Wild	Herbivorous	22001801632017	Illumina HiSeq	Levin et al.(2021)
22001801632018	Kobus kob	Wild	Herbivorous	22001801632018	Illumina HiSeq	Levin et al.(2021)

22001801632020	Loxodonta africana	Wild	Herbivorous	22001801632020	Illumina HiSeq	Levin et al.(2021)
22001801632025	Kobus ellipsiprymnus	Wild	Herbivorous	22001801632025	Illumina HiSeq	Levin et al.(2021)
22001801632032	Giraffa camelopardalis	Wild	Herbivorous	22001801632032	Illumina HiSeq	Levin et al.(2021)
22001801632038	Kobus kob	Wild	Herbivorous	22001801632038	Illumina HiSeq	Levin et al.(2021)
22001801632039	Loxodonta africana	Wild	Herbivorous	22001801632039	Illumina HiSeq	Levin et al.(2021)
22001801632046	Syncerus caffer	Wild	Herbivorous	22001801632046	Illumina HiSeq	Levin et al.(2021)
22001801632049	Kobus ellipsiprymnus	Wild	Herbivorous	22001801632049	Illumina HiSeq	Levin et al.(2021)
22001801632050	Alcelaphus buselaphus	Wild	Herbivorous	22001801632050	Illumina HiSeq	Levin et al.(2021)
22001801632051	Aepyceros melampus	Wild	Herbivorous	22001801632051	Illumina HiSeq	Levin et al.(2021)
22001801632071	Hippopotamus amphibius	Wild	Herbivorous	22001801632071	Illumina HiSeq	Levin et al.(2021)
22001801632074	Ourebia ourebi	Wild	Herbivorous	22001801632074	Illumina HiSeq	Levin et al.(2021)
22001801632075	Damaliscus lunatus	Wild	Herbivorous	22001801632075	Illumina HiSeq	Levin et al.(2021)
22001801632120	Ceratotherium simum	Wild	Herbivorous	22001801632120	Illumina HiSeq	Levin et al.(2021)
22001801632121	Alcelaphus buselaphus	Wild	Herbivorous	22001801632121	Illumina HiSeq	Levin et al.(2021)
22001801632122	Syncerus caffer	Wild	Herbivorous	22001801632122	Illumina HiSeq	Levin et al.(2021)
22001801632129	Piliocolobus tephrosceles	Wild	Herbivorous	22001801632129	Illumina HiSeq	Levin et al.(2021)
22001801632180	Hippopotamus amphibius	Wild	Herbivorous	22001801632180	Illumina HiSeq	Levin et al.(2021)
22001801632182	Colobus guereza	Wild	Herbivorous	22001801632182	Illumina HiSeq	Levin et al.(2021)
22001801632188	Pan troglodytes	Wild	Herbivorous	22001801632188	Illumina HiSeq	Levin et al.(2021)
22001801632189	Loxodonta africana	Wild	Herbivorous	22001801632189	Illumina HiSeq	Levin et al.(2021)
22001801632193	Loxodonta africana	Wild	Herbivorous	22001801632193	Illumina HiSeq	Levin et al.(2021)
22001801632204	Colobus guereza	Wild	Herbivorous	22001801632204	Illumina HiSeq	Levin et al.(2021)
22001801632205	Aepyceros melampus	Wild	Herbivorous	22001801632205	Illumina HiSeq	Levin et al.(2021)
22001801632207	Loxodonta cyclotis	Wild	Herbivorous	22001801632207	Illumina HiSeq	Levin et al.(2021)
22001801632211	Phacochoerus africanus	Wild	Herbivorous	22001801632211	Illumina HiSeq	Levin et al.(2021)
22001801632214	Kobus kob	Wild	Herbivorous	22001801632214	Illumina HiSeq	Levin et al.(2021)
22001801632217	Piliocolobus tephrosceles	Wild	Herbivorous	22001801632217	Illumina HiSeq	Levin et al.(2021)
22001801632221	Prolemur simus	Wild	Herbivorous	22001801632221	Illumina HiSeq	Levin et al.(2021)
22001801632223	Papio anubis	Wild	Herbivorous	22001801632223	Illumina HiSeq	Levin et al.(2021)
22001801632226	Pan troglodytes	Wild	Herbivorous	22001801632226	Illumina HiSeq	Levin et al.(2021)
22001801632235	Piliocolobus tephrosceles	Wild	Herbivorous	22001801632235	Illumina HiSeq	Levin et al.(2021)
22001801632285	Equus burchellii	Wild	Herbivorous	22001801632285	Illumina HiSeq	Levin et al.(2021)
22001801632288	Damaliscus lunatus	Wild	Herbivorous	22001801632288	Illumina HiSeq	Levin et al.(2021)
22001803500793	Mirounga leonina	Wild	Herbivorous	22001803500793	Illumina HiSeq	Levin et al.(2021)
22001803500803	Mirounga leonina	Wild	Herbivorous	22001803500803	Illumina HiSeq	Levin et al.(2021)
22001803501039	Oryctolagus cuniculus	Wild	Herbivorous	22001803501039	Illumina HiSeq	Levin et al.(2021)
22001803501041	Balaenoptera borealis	Wild	Herbivorous	22001803501041	Illumina HiSeq	Levin et al.(2021)
32001806570020	Macropus giganteus	Wild	Herbivorous	32001806570020	Illumina HiSeq	Levin et al.(2021)
32001806570028	Macropus giganteus	Wild	Herbivorous	32001806570028	Illumina HiSeq	Levin et al.(2021)
32001806570051	Notamacropus parryi	Wild	Herbivorous	32001806570051	Illumina HiSeq	Levin et al.(2021)
32001806570074	Pseudomys gracilicaudatus	Wild	Herbivorous	32001806570074	Illumina HiSeq	Levin et al.(2021)
32001806570094	Pseudomys gracilicaudatus	Wild	Herbivorous	32001806570094	Illumina HiSeq	Levin et al.(2021)
32001806570114	Notamacropus parryi	Wild	Herbivorous	32001806570114	Illumina HiSeq	Levin et al.(2021)
32001806570319	Trichosurus vulpecula	Wild	Herbivorous	32001806570319	Illumina HiSeq	Levin et al.(2021)

32001806570322	Macropus giganteus	Wild	Herbivorous	32001806570322	Illumina HiSeq	Levin et al.(2021)
32001806570325	Antechinus flavipes	Wild	Herbivorous	32001806570325	Illumina HiSeq	Levin et al.(2021)
32001806570326	Pseudomys gracilicaudatus	Wild	Herbivorous	32001806570326	Illumina HiSeq	Levin et al.(2021)
32001806570328	Trichosurus vulpecula	Wild	Herbivorous	32001806570328	Illumina HiSeq	Levin et al.(2021)
32001806570329	Trichosurus vulpecula	Wild	Herbivorous	32001806570329	Illumina HiSeq	Levin et al.(2021)
32001806570343	Pseudomys gracilicaudatus	Wild	Herbivorous	32001806570343	Illumina HiSeq	Levin et al.(2021)
32001806570352	Antechinus flavipes	Wild	Herbivorous	32001806570352	Illumina HiSeq	Levin et al.(2021)
11641712540072	Potamochoerus larvatus	Wild	Ominivorous	11641712540072	Illumina HiSeq	Levin et al.(2021)
32001806570009	Trichosurus vulpecula	Wild	Ominivorous	32001806570009	Illumina HiSeq	Levin et al.(2021)
22001801632197	Phacochoerus africanus	Wild	Ominivorous	22001801632197	Illumina HiSeq	Levin et al.(2021)
22001801632166	Papio anubis	Wild	Ominivorous	22001801632166	Illumina HiSeq	Levin et al.(2021)
22001801632173	Papio anubis	Wild	Ominivorous	22001801632173	Illumina HiSeq	Levin et al.(2021)
22001801632009	Pan troglodytes	Wild	Ominivorous	22001801632009	Illumina HiSeq	Levin et al.(2021)
22001801631175	Microcebus murinus	Wild	Ominivorous	22001801631175	Illumina HiSeq	Levin et al.(2021)
22001708570027	Herpestes ichneumon	Wild	Ominivorous	22001708570027	Illumina HiSeq	Levin et al.(2021)
22001801631190	Hapalemur aureus	Wild	Ominivorous	22001801631190	Illumina HiSeq	Levin et al.(2021)
11641712540151	Eulemur rufifrons	Wild	Ominivorous	11641712540151	Illumina HiSeq	Levin et al.(2021)
11641712540156	Cryptoprocta ferox	Wild	Carnivorous	11641712540156	Illumina HiSeq	Levin et al.(2021)
32001806570103	Planigale maculata	Wild	Carnivorous	32001806570103	Illumina HiSeq	Levin et al.(2021)
32001806570107	Antechinus flavipes	Wild	Carnivorous	32001806570107	Illumina HiSeq	Levin et al.(2021)
22001803501051	Mirounga leonina	Wild	Carnivorous	22001803501051	Illumina HiSeq	Levin et al.(2021)
22001803502283	Balaenoptera borealis	Wild	Carnivorous	22001803502283	Illumina HiSeq	Levin et al.(2021)
22001803502410	Balaenoptera borealis	Wild	Carnivorous	22001803502410	Illumina HiSeq	Levin et al.(2021)
22001803502412	Otaria byronia	Wild	Carnivorous	22001803502412	Illumina HiSeq	Levin et al.(2021)
22001803502414	Otaria byronia	Wild	Carnivorous	22001803502414	Illumina HiSeq	Levin et al.(2021)
22001803502427	Otaria byronia	Wild	Carnivorous	22001803502427	Illumina HiSeq	Levin et al.(2021)
22001803500824	Arctocephalus australis	Wild	Carnivorous	22001803500824	Illumina HiSeq	Levin et al.(2021)
22001801632239	Miniopterus manavi	Wild	Carnivorous	22001801632239	Illumina HiSeq	Levin et al.(2021)
22001801632265	Miniopterus manavi	Wild	Carnivorous	22001801632265	Illumina HiSeq	Levin et al.(2021)
22001708574871	Hyaena hyaena	Wild	Carnivorous	22001708574871	Illumina HiSeq	Levin et al.(2021)
22001708574884	Erinaceus europaeus	Wild	Carnivorous	22001708574884	Illumina HiSeq	Levin et al.(2021)
