# Supplementary material for: Pseudomonas-associated bacteria play a key role in obtaining nutrition from bamboo for the giant panda (Ailuropoda melanoleuca)
Source: Microbiol Spectr. 2024 Feb 2;12(3):e03819-23. doi: 10.1128/spectrum.03819-23 (PMC10913395; doi:10.1128/spectrum.03819-23)
Supplement: Table S3 — Statistics on the taxonomic assignment of enzymes involved in degrading lignin derivatives from wild giant pandas. [file spectrum.03819-23-s0007.pdf]

Table S3. Statistics on the taxonomic assignment of enzymes involved in degrading lignin derivatives from wild giant pandas.

Gene name	Number of identified lineage (genus)	Number of all identified genes	Number of genes from *Pseudomonas*- associated bacteria	The Proportion of *Pseudomonas*-associated bacteria (%)
Fcs	52	137	20	14.60
ferB	21	32	7	21.88
vdh	5	12	8	66.67
pobA	70	152	31	20.39
vanA	19	54	16	29.63
vanB	21	60	17	28.33
catA	35	97	30	33.33
catB	39	94	23	24.47
catC	24	48	13	27.08
pcaD	50	163	38	23.31
pcaL	3	4	2	50
pcaI	33	60	12	20
pcaJ	46	90	15	16.67
pcaG	31	74	22	29.73
pcaH	45	69	12	17.4
pcaC	95	295	48	16.27
pcaF	5	8	3	37.5
HPD	71	300	52	17.33
paaE	55	55	9	16.36
HGD	87	199	28	14.08
maiA	19	32	7	21.88
FAH	50	163	38	23.31
faaH	89	158	29	18.35
