# Supplementary material for: Pseudomonas-associated bacteria play a key role in obtaining nutrition from bamboo for the giant panda (Ailuropoda melanoleuca)
Source: Microbiol Spectr. 2024 Feb 2;12(3):e03819-23. doi: 10.1128/spectrum.03819-23 (PMC10913395; doi:10.1128/spectrum.03819-23)
Supplement: Table S4 — Information of individual draft genomes recovered from the gut metagenomic data of wild giant pandas in this study. [file spectrum.03819-23-s0008.pdf]

Table S4. The information of individual draft genomes (bins) which were recovered from the gut metagenomic data of wild giant pandas in this study.

Binning ID	Completeness	Contamination	Ratio of GC	Lineage	N50	Size
Bin1	96.09	1.366	0.566	*Pseudomonas*	33840	4,755,608
Bin2	71.13	9.905	0.637	*Microbacteriaceae*	1806	3,155,662
Bin3	90.47	1.12	0.447	*Cellvibrionaceae*	10371	3,579,560
Bin4	95.47	0.331	0.382	*Lysinibacillus*	5543	3,043,211
Bin5	100	0.374	0.371	*Streptococcaceae*	115072	2,099,471
Bin6	96.83	1.304	0.496	*Rhizobiales*	17202	3,917,148
Bin7	96.68	0.15	0.436	*Leuconostoc*	7333	1,544,251
Bin8	95.52	1.7	0.637	*Microterricola viridarii*	15277	3,579,366
Bin9	80.5	3.165	0.624	*Achromobacter*	7414	5,436,964
Bin10	71.57	2.506	0.34	*Flavobacterium*	4431	4,132,461
Bin11	78.16	5.282	0.672	*Stenotrophomonas sp. LM091*	4275	3,521,650
Bin12	95.02	4.038	0.577	*Comamonas*	24856	3,627,295
Bin13	96.87	1.361	0.491	*Hafniaceae*	16163	4,077,809
Bin14	76.45	0.977	0.625	*Oxalobacteraceae*	14915	5,401,556
Bin15	85.31	4.587	0.322	*Flavobacterium sp. 140616W15*	13403	5,637,583
Bin16	95.49	0.53	0.542	*Enterobacteriaceae*	37594	4,950,555
Bin17	96.94	3.713	0.608	*Pseudomonas*	24099	5,847,377
Bin18	93.8	1.825	0.359	*Sphingobacterium sp. PM2-P1-29*	14387	4,603,855
Bin19	99.33	0.551	0.383	*Lysinibacillus*	13929	3,999,274
Bin20	90.09	2.154	0.473	*Yersinia*	7831	3,782,573
Bin21	87.19	2.764	0.604	*Cutibacterium acnes*	3536	2,051,155
Bin22	97.04	0.976	0.282	*Clostridium*	21861	3,547,461
Bin23	94.44	0.476	0.363	*Sphingobacterium*	24452	5,013,028
Bin24	98.39	0.699	0.587	*Alcaligenaceae*	238204	5,069,453
Bin25	99.62	0	0.47	*Yersiniaceae*	126430	4,000,426
Bin26	83.46	1.764	0.643	*Janthinobacterium*	18631	5,670,813
Bin27	86.53	1.287	0.339	*Flavobacterium*	11180	4,574,008
Bin28	78.26	2.008	0.439	*Leuconostoc*	3321	1,178,185
Bin29	90.71	1.728	0.658	*Comamonadaceae*	15638	5,414,517
Bin30	85.34	1.198	0.531	*Rahnella*	35326	4,327,131
Bin31	96.3	2.509	0.707	*Janibacter*	6538	3,159,660
Bin32	90.91	1.062	0.438	*Leuconostoc*	10476	1,413,548
Bin33	84.16	3.138	0.586	*Pseudomonas*	16014	4,776,869
Bin34	79.74	3.014	0.593	*Arthrobacter*	2604	3,931,345
Bin35	87.3	3.059	0.706	*Janibacter*	5886	2,984,401
Bin36	92.65	3.165	0.275	*Clostridium*	6186	1,874,850
Bin37	99.5	1.498	0.403	*Streptococcaceae*	51489	2,279,131
Bin38	94.9	2.309	0.371	*Streptococcus pasteurianus*	21596	2,004,371
Bin39	73.27	3.448	0.545	*Enterobacteriaceae*	8924	4,311,391
Bin40	86.05	3.699	0.576	*Comamonas*	4749	3,022,187
Bin41	98.34	3.537	0.378	*Lysinibacillus*	5857	3,676,956
Bin42	95.39	1.228	0.601	*Cutibacterium acnes*	5151	2,298,473

Bin43	71.05	3.448	0.364	*Sphingobacterium sp. PM2-P1-29*	4440	4,806,346
Bin44	73.73	2.586	0.622	*Achromobacter*	8692	5,860,968
Bin45	99.84	0	0.587	*Alcaligenaceae*	188113	5,092,273
Bin46	95.43	1.612	0.283	*Clostridiaceae*	40953	2,723,232
Bin47	80.67	3.692	0.304	*Helicobacteraceae*	3843	1,667,016
Bin48	88.24	2.93	0.283	*Clostridium*	10382	2,981,146
Bin49	71.85	4.634	0.636	*Microbacteriaceae*	3039	2,521,974
Bin50	87.72	1.797	0.446	*Cellvibrionaceae*	12718	3,477,199
Bin51	85.3	4.32	0.615	*Oxalobacteraceae*	11315	6,169,746
Bin52	96.4	1.25	0.58	*Pseudomonas fluorescens PfO-1*	12264	4,982,434
