# Supplementary material for: Pseudomonas-associated bacteria play a key role in obtaining nutrition from bamboo for the giant panda (Ailuropoda melanoleuca)
Source: Microbiol Spectr. 2024 Feb 2;12(3):e03819-23. doi: 10.1128/spectrum.03819-23 (PMC10913395; doi:10.1128/spectrum.03819-23)
Supplement: Table S5 — Genes involved in lignin degradation identified in the bins retrieved from wild giant pandas. [file spectrum.03819-23-s0009.pdf]

Table S5.Genes involved in lignin degradation identified in the Bins which were retrieved from wild giant pandas.

Bin	Contig	Function	Subsystem
Bin 1, Bin 17, Bin 33, and Bin 52	k141_1477432_length_5419_cov_846.0034	Catechol 1,2-dioxygenase (EC 1.13.11.1)	Catechol branch of beta-ketoadipate pathway
	k141_1477432_length_5419_cov_846.0034	Muconolactone isomerase (EC 5.3.3.4)	Catechol branch of beta-ketoadipate pathway
	k141_246360_length_25748_cov_825.0000	Succinyl-CoA:3-ketoacid-coenzyme A transferase subunit A (EC 2.8.3.5)	Catechol branch of beta-ketoadipate pathway; Leucine Degradation and HMG-CoA Metabolism; Protocatechuate branch of beta-ketoadipate pathway
	k141_246360_length_25748_cov_825.0000	Succinyl-CoA:3-ketoacid-coenzyme A transferase subunit B (EC 2.8.3.5)	Catechol branch of beta-ketoadipate pathway; Leucine Degradation and HMG-CoA Metabolism; Protocatechuate branch of beta-ketoadipate pathway
	k141_432594_length_9046_cov_822.0000	Succinyl-CoA:3-ketoacid-coenzyme A transferase subunit A (EC 2.8.3.5)	Catechol branch of beta-ketoadipate pathway; Leucine Degradation and HMG-CoA Metabolism; Protocatechuate branch of beta-ketoadipate pathway
	k141_432594_length_9046_cov_822.0000	Succinyl-CoA:3-ketoacid-coenzyme A transferase subunit B (EC 2.8.3.5)	Catechol branch of beta-ketoadipate pathway; Leucine Degradation and HMG-CoA Metabolism; Protocatechuate branch of beta-ketoadipate pathway
	k141_43825_length_6761_cov_875.0000	Muconate cycloisomerase (EC 5.5.1.1)	Catechol branch of beta-ketoadipate pathway; Muconate lactonizing enzyme family
	k141_799824_length_10966_cov_827.0000	3-oxoadipate CoA-transferase subunit A (EC 2.8.3.6)	Catechol branch of beta-ketoadipate pathway; Protocatechuate branch of beta-ketoadipate pathway
	k141_799824_length_10966_cov_827.0000	3-oxoadipate CoA-transferase subunit B (EC 2.8.3.6)	Catechol branch of beta-ketoadipate pathway; Protocatechuate branch of beta-ketoadipate pathway
	k141_799824_length_10966_cov_827.0000	Beta-ketoadipate enol-lactone hydrolase (EC 3.1.1.24)	Catechol branch of beta-ketoadipate pathway; Protocatechuate branch of beta-ketoadipate pathway
	k141_799824_length_10966_cov_827.0000	3-oxoadipyl-CoA thiolase (EC 2.3.1.174)	Catechol branch of beta-ketoadipate pathway; Leucine Degradation and HMG-CoA Metabolism; Protocatechuate branch of beta-ketoadipate pathway
	k141_1550194_length_14335_cov_848.0000	Protocatechuate 3,4-dioxygenase alpha chain (EC 1.13.11.3)	Protocatechuate branch of beta-ketoadipate pathway
	k141_1550194_length_14335_cov_848.0000	Protocatechuate 3,4-dioxygenase beta chain (EC 1.13.11.3)	Protocatechuate branch of beta-ketoadipate pathway
	k141_2114240_length_8040_cov_833.2317	4-carboxymuconolactone decarboxylase (EC 4.1.1.44)	Protocatechuate branch of beta-ketoadipate pathway
	k141_799824_length_10966_cov_827.0000	Pca regulon regulatory protein PcaR	Protocatechuate branch of beta-ketoadipate pathway
	k141_799824_length_10966_cov_827.0000	dicarboxylic acid transporter PcaT	Protocatechuate branch of beta-ketoadipate pathway
	k141_799824_length_10966_cov_827.0000	3-carboxy-cis,cis-muconate cycloisomerase (EC 5.5.1.2)	Protocatechuate branch of beta-ketoadipate pathway
	k141_799824_length_10966_cov_827.0000	4-carboxymuconolactone decarboxylase (EC 4.1.1.44)	Protocatechuate branch of beta-ketoadipate pathway
	k141_981091_length_5373_cov_622.0000	4-carboxymuconolactone decarboxylase (EC 4.1.1.44)	Protocatechuate branch of beta-ketoadipate pathway
	k141_1688534_length_9870_cov_911.0000	Transcriptional regulator, IclR family	Homogentisate pathway of aromatic compound degradation
	k141_1921086_length_38366_cov_824.4826	Transcriptional regulator, IclR family	Homogentisate pathway of aromatic compound degradation
	k141_2019723_length_17172_cov_69.0000	Transcriptional regulator, IclR family	Homogentisate pathway of aromatic compound degradation
	k141_246360_length_25748_cov_825.0000	Transcriptional regulator, IclR family	Homogentisate pathway of aromatic compound degradation
	k141_1051773_length_29758_cov_837.0000	Biosynthetic Aromatic amino acid aminotransferase alpha (EC 2.6.1.57)	Homogentisate pathway of aromatic compound degradation; Phenylalanine and Tyrosine Branches from Chorismate; Pterin carbinolamine dehydratase
	k141_1618549_length_19774_cov_872.0000	Homogentisate 1,2-dioxygenase (EC 1.13.11.5)	Homogentisate pathway of aromatic compound degradation; Pterin carbinolamine dehydratase
	k141_1618549_length_19774_cov_872.0000	Fumarylacetoacetase (EC 3.7.1.2)	Homogentisate pathway of aromatic compound degradation; Pterin carbinolamine dehydratase

k141_1618549_length_19774_cov_872.0000	Maleylacetoacetate isomerase (EC 5.2.1.2) @ Glutathione S-transferase, zeta (EC 2.5.1.18)	Gentisate degradation; Glutathione: Non-redox reactions; Homogentisate pathway of aromatic compound degradation; Pterin carbinolamine dehydratase
k141_1550194_length_14335_cov_848.0000	4-hydroxyphenylpyruvate dioxygenase (EC 1.13.11.27)	Aromatic amino acid degradation; Homogentisate pathway of aromatic compound degradation; Pterin carbinolamine dehydratase
k141_609503_length_3895_cov_745.0000	4-hydroxyphenylpyruvate dioxygenase (EC 1.13.11.27)	Aromatic amino acid degradation; Homogentisate pathway of aromatic compound degradation; Pterin carbinolamine dehydratase
k141_1912767_length_4780_cov_800.2240	Aspartate aminotransferase (EC 2.6.1.1) @ Aromatic-amino-acid aminotransferase (EC 2.6.1.57)	CBSS-216591.1.peg.168; Glutamine, Glutamate, Aspartate and Asparagine Biosynthesis; Homogentisate pathway of aromatic compound degradation; Pterin carbinolamine dehydratase; Threonine and Homoserine Biosynthesis
k141_1106069_length_22706_cov_870.5268	P-hydroxybenzoate hydroxylase (EC 1.14.13.2)	p-Hydroxybenzoate degradation
k141_2019723_length_17172_cov_69.0000	Vanillate O-demethylase oxygenase subunit (EC 1.14.13.82)	none
k141_2019723_length_17172_cov_69.0000	Flavodoxin reductases (ferredoxin-NADPH reductases) family 1; Vanillate O-demethylase oxidoreductase (EC 1.14.13.-)	Anaerobic respiratory reductases; Anaerobic respiratory reductases
k141_1873074_length_23330_cov_864.0497	Aldehyde dehydrogenase (EC 1.2.1.3); Probable coniferyl aldehyde dehydrogenase (EC 1.2.1.68)	Methylglyoxal Metabolism; Methylglyoxal Metabolism
k141_540920_length_6961_cov_823.8504	Multicopper oxidase	Copper homeostasis
k141_1045062_length_4277_cov_878.5909	FIG00003370: Multicopper polyphenol oxidase	none
k141_1153133_length_14502_cov_866.0000	Putative multicopper oxidases	none
k141_717462_length_43354_cov_27.9181	Succinyl-CoA:3-ketoacid-coenzyme A transferase subunit B (EC 2.8.3.5)	Branched chain amino acid degradation regulons;Catechol branch of beta-ketoadipate pathway;Leucine Degradation and HMG-CoA Metabolism;Protocatechuate branch of beta-ketoadipate pathway;Serine-glyoxylate cycle
k141_717462_length_43354_cov_27.9181	Succinyl-CoA:3-ketoacid-coenzyme A transferase subunit A (EC 2.8.3.5)	Branched chain amino acid degradation regulons;Catechol branch of beta-ketoadipate pathway;Leucine Degradation and HMG-CoA Metabolism;Protocatechuate branch of beta-ketoadipate pathway;Serine-glyoxylate cycle
k141_1030602_length_4153_cov_23.0000	Muconolactone isomerase (EC 5.3.3.4)	Catechol branch of beta-ketoadipate pathway
k141_1070080_length_8886_cov_15.0000	mandelate racemase/muconate lactonizing enzyme family protein	Catechol branch of beta-ketoadipate pathway
k141_346406_length_1650_cov_54.0000	mandelate racemase/muconate lactonizing enzyme family protein	Catechol branch of beta-ketoadipate pathway
k141_1546098_length_5871_cov_25.0000	Muconate cycloisomerase (EC 5.5.1.1)	Catechol branch of beta-ketoadipate pathway;Muconate lactonizing enzyme family
k141_1792833_length_8973_cov_83.0000	Muconate cycloisomerase (EC 5.5.1.1)	Catechol branch of beta-ketoadipate pathway;Muconate lactonizing enzyme family
k141_1817607_length_7918_cov_29.0000	Acetyl-CoA acetyltransferase (EC 2.3.1.9) @ 3-oxoadipyl-CoA thiolase (EC 2.3.1.174)	Acetyl-CoA fermentation to Butyrate;Butanol Biosynthesis;Butyrate metabolism cluster;Polyhydroxybutyrate metabolism;Serine-glyoxylate cycle
k141_1030602_length_4153_cov_23.0000	3-oxoadipate CoA-transferase subunit B (EC 2.8.3.6)	Catechol branch of beta-ketoadipate pathway;Protocatechuate branch of beta-ketoadipate pathway
k141_1030602_length_4153_cov_23.0000	Beta-ketoadipate enol-lactone hydrolase (EC 3.1.1.24)	Catechol branch of beta-ketoadipate pathway;Protocatechuate branch of beta-ketoadipate pathway
k141_1713974_length_3956_cov_103.0000	3-oxoadipate CoA-transferase subunit A (EC 2.8.3.6)	Catechol branch of beta-ketoadipate pathway;Protocatechuate branch of beta-ketoadipate pathway
k141_1713974_length_3956_cov_103.0000	3-oxoadipate CoA-transferase subunit B (EC 2.8.3.6)	Catechol branch of beta-ketoadipate pathway;Protocatechuate branch of beta-ketoadipate pathway
k141_2026656_length_14161_cov_46.0000	3-oxoadipate CoA-transferase subunit B (EC 2.8.3.6)	Catechol branch of beta-ketoadipate pathway;Protocatechuate branch of beta-ketoadipate pathway
k141_2026656_length_14161_cov_46.0000	3-oxoadipate CoA-transferase subunit A (EC 2.8.3.6)	Catechol branch of beta-ketoadipate pathway;Protocatechuate branch of beta-ketoadipate pathway

Bin 9 and Bin 44	k141_703718_length_4334_cov_20.0000	Beta-ketoadipate enol-lactone hydrolase (EC 3.1.1.24)	Catechol branch of beta-ketoadipate pathway;Protocatechuate branch of beta-ketoadipate pathway
	k141_908554_length_5419_cov_19.0000	3-oxoadipate CoA-transferase subunit A (EC 2.8.3.6)	Catechol branch of beta-ketoadipate pathway;Protocatechuate branch of beta-ketoadipate pathway
	k141_962908_length_20715_cov_90.0000	3-oxoadipate CoA-transferase subunit A (EC 2.8.3.6)	Catechol branch of beta-ketoadipate pathway;Protocatechuate branch of beta-ketoadipate pathway
	k141_962908_length_20715_cov_90.0000	3-oxoadipate CoA-transferase subunit B (EC 2.8.3.6)	Catechol branch of beta-ketoadipate pathway;Protocatechuate branch of beta-ketoadipate pathway
	k141_1031727_length_5979_cov_13.0000	3-carboxy-cis,cis-muconate cycloisomerase (EC 5.5.1.2)	Protocatechuate branch of beta-ketoadipate pathway
	k141_1031727_length_5979_cov_13.0000	Protocatechuate 3,4-dioxygenase alpha chain (EC 1.13.11.3)	Protocatechuate branch of beta-ketoadipate pathway
	k141_1031727_length_5979_cov_13.0000	Protocatechuate 3,4-dioxygenase beta chain (EC 1.13.11.3)	Protocatechuate branch of beta-ketoadipate pathway
	k141_1190964_length_4746_cov_25.2780	4-carboxymuconolactone decarboxylase (EC 4.1.1.44)	Protocatechuate branch of beta-ketoadipate pathway
	k141_1219445_length_11005_cov_20.0000	4-carboxymuconolactone decarboxylase (EC 4.1.1.44)	Protocatechuate branch of beta-ketoadipate pathway
	k141_2026656_length_14161_cov_46.0000	Pca regulon regulatory protein PcaR	Protocatechuate branch of beta-ketoadipate pathway
	k141_908554_length_5419_cov_19.0000	Pca regulon regulatory protein PcaR	Protocatechuate branch of beta-ketoadipate pathway
	k141_37474_length_3721_cov_29.0000	4-hydroxyphenylpyruvate dioxygenase (EC 1.13.11.27)	Aromatic amino acid degradation;Homogentisate pathway of aromatic compound degradation;Pterin carbinolamine dehydratase
	k141_37474_length_3721_cov_29.0000	4-hydroxyphenylpyruvate dioxygenase (EC 1.13.11.27)	Aromatic amino acid degradation;Homogentisate pathway of aromatic compound degradation;Pterin carbinolamine dehydratase
	k141_1622536_length_12434_cov_25.0000	Maleylacetoacetate isomerase (EC 5.2.1.2) @ Glutathione S-transferase, zeta (EC 2.5.1.18)	Gentisate degradation;Glutathione: Non-redox reactions;Homogentisate pathway of aromatic compound degradation;Pterin carbinolamine dehydratase;Salicylate and gentisate catabolism
	k141_1020647_length_2997_cov_8.0000	Transcriptional regulator, IclR family	Homogentisate pathway of aromatic compound degradation
	k141_1068200_length_7877_cov_42.0000	Transcriptional regulator, IclR family	Homogentisate pathway of aromatic compound degradation
	k141_1133711_length_12490_cov_25.6012	Transcriptional regulator, IclR family	Homogentisate pathway of aromatic compound degradation
	k141_1219445_length_11005_cov_20.0000	Transcriptional regulator, IclR family	Homogentisate pathway of aromatic compound degradation
	k141_1243267_length_21281_cov_34.8890	Transcriptional regulator, IclR family	Homogentisate pathway of aromatic compound degradation
	k141_1298122_length_20665_cov_31.0000	Transcriptional regulator, IclR family	Homogentisate pathway of aromatic compound degradation
	k141_1468158_length_10402_cov_20.0000	Transcriptional regulator, IclR family	Homogentisate pathway of aromatic compound degradation
	k141_1513523_length_9957_cov_48.0000	Transcriptional regulator, IclR family	Homogentisate pathway of aromatic compound degradation
	k141_1680874_length_11015_cov_34.0000	Transcriptional regulator, IclR family	Homogentisate pathway of aromatic compound degradation
	k141_1720834_length_6885_cov_17.0000	Transcriptional regulator, IclR family	Homogentisate pathway of aromatic compound degradation
	k141_1722443_length_10705_cov_20.7974	Transcriptional regulator, IclR family	Homogentisate pathway of aromatic compound degradation
	k141_1792833_length_8973_cov_83.0000	Transcriptional regulator, IclR family	Homogentisate pathway of aromatic compound degradation
	k141_1878766_length_3765_cov_21.0000	Transcriptional regulator, IclR family	Homogentisate pathway of aromatic compound degradation
	k141_1910136_length_27322_cov_35.8314	Transcriptional regulator, IclR family	Homogentisate pathway of aromatic compound degradation
	k141_1937970_length_2651_cov_11.4171	Transcriptional regulator, IclR family	Homogentisate pathway of aromatic compound degradation
	k141_2015188_length_4686_cov_30.0000	Transcriptional regulator, IclR family	Homogentisate pathway of aromatic compound degradation
	k141_2107888_length_12855_cov_37.0000	Transcriptional regulator, IclR family	Homogentisate pathway of aromatic compound degradation

	k141_2274000_length_7756_cov_34.0000	Transcriptional regulator, IclR family	Homogentisate pathway of aromatic compound degradation
	k141_490917_length_26748_cov_27.0000	Transcriptional regulator, IclR family	Homogentisate pathway of aromatic compound degradation
	k141_514666_length_6667_cov_17.0000	Transcriptional regulator, IclR family	Homogentisate pathway of aromatic compound degradation
	k141_528544_length_8939_cov_32.0000	Transcriptional regulator, IclR family	Homogentisate pathway of aromatic compound degradation
	k141_840621_length_6135_cov_33.0000	Transcriptional regulator, IclR family	Homogentisate pathway of aromatic compound degradation
	k141_962908_length_20715_cov_90.0000	Transcriptional regulator, IclR family	Homogentisate pathway of aromatic compound degradation
	k141_1581435_length_9717_cov_44.0000	Aromatic-amino-acid aminotransferase (EC 2.6.1.57)	Homogentisate pathway of aromatic compound degradation;Pterin carbinolamine dehydratase
	k141_654353_length_11771_cov_25.0000	Homogentisate 1,2-dioxygenase (EC 1.13.11.5)	Homogentisate pathway of aromatic compound degradation;Pterin carbinolamine dehydratase
	k141_936897_length_13071_cov_43.0000	Aromatic-amino-acid aminotransferase (EC 2.6.1.57)	Homogentisate pathway of aromatic compound degradation;Pterin carbinolamine dehydratase
	k141_654353_length_11771_cov_25.0000	Fumarylacetoacetase (EC 3.7.1.2)	Homogentisate pathway of aromatic compound degradation;Pterin carbinolamine dehydratase;Salicylate and gentisate catabolism
	k141_1309965_length_4077_cov_15.7370	Aspartate aminotransferase (AspB-4) (EC 2.6.1.1)	Homogentisate pathway of aromatic compound degradation;Pterin carbinolamine dehydratase;Salicylate and gentisate catabolism
	k141_1466370_length_13771_cov_26.0000	Multicopper oxidase	Copper homeostasis
	k141_352801_length_15028_cov_30.0000	FIG00003370: Multicopper polyphenol oxidase	none
