# Supplementary material for: Pseudomonas-associated bacteria play a key role in obtaining nutrition from bamboo for the giant panda (Ailuropoda melanoleuca)
Source: Microbiol Spectr. 2024 Feb 2;12(3):e03819-23. doi: 10.1128/spectrum.03819-23 (PMC10913395; doi:10.1128/spectrum.03819-23)
Supplement: Table S6 — Taxonomic assignment of laccase-like multicopper oxidase genes in the feces of wild giant pandas. [file spectrum.03819-23-s0010.pdf]

Table S6. The taxonomic assignment of laccase-like multicopper oxidase genes in the feces of wild giant pandas.

Clone ID	Sample ID	The closest neighbors in NCBI	The similarity (%) with closest neighbors	Gene Length (bp)
Clone_1	WP5	*Acinetobacter* sp	86	162
Clone_2	WP10	*Acinetobacter* sp	47	143
Clone_3	WP14	*Acinetobacter* sp	93	142
Clone_4	WP5	*Brevundimonas abyssalis*	85	143
Clone_5	WP10	*Brevundimonas abyssalis*	85	143
Clone_6	WP14	*Brevundimonas diminuta*	93	141
Clone_7	WP5	*Brevundimonas diminuta*	95	141
Clone_8	WP14	*Brevundimonas diminuta*	91	142
Clone_9	WP10	*Brevundimonas naejangsanensis*	95	142
Clone_10	WP14	*Brevundimonas naejangsanensis*	94	142
Clone_11	WP5	*Caulobacter* sp.	93	141
Clone_12	WP10	*Flavobacterium* sp	94	142
Clone_13	WP5	*Flavobacterium* sp	95	141
Clone_14	WP14	*Flavobacterium* sp. URHB0058	93	142
Clone_15	WP10	*Flavobacterium* sp. URHB0058	95	142
Clone_16	WP5	*Janthinobacterium*	94	134
Clone_17	WP14	*Klebsiella* sp	96	143
Clone_18	WP10	*Klebsiella* sp	94	143
Clone_19	WP5	*Pseudomonas putida*	91	142
Clone_20	WP5	*Pseudomonas putida*	94	142
Clone_21	WP5	*Pseudomonas putida*	94	141
Clone_22	WP5	*Pseudomonas putida*	95	141
Clone_23	WP5	*Pseudomonas putida*	89	142
Clone_24	WP5	*Pseudomonas putida*	91	142
Clone_25	WP5	*Pseudomonas putida*	95	142
Clone_26	WP5	*Pseudomonas putida*	96	142
Clone_27	WP5	*Pseudomonas putida*	91	142
Clone_28	WP5	*Pseudomonas putida*	89	143
Clone_29	WP5	*Pseudomonas putida*	96	143
Clone_30	WP5	*Pseudomonas putida*	93	142
Clone_31	WP5	*Pseudomonas putida*	96	142
Clone_32	WP5	*Pseudomonas putida*	96	142
Clone_33	WP5	*Pseudomonas putida*	91	142
Clone_34	WP5	*Pseudomonas putida*	89	148
Clone_35	WP5	*Pseudomonas putida*	96	142
Clone_36	WP10	*Pseudomonas putida*	94	142
Clone_37	WP10	*Pseudomonas putida*	98	141
Clone_38	WP10	*Pseudomonas putida*	98	141
Clone_39	WP10	*Pseudomonas putida*	98	141
Clone_40	WP10	*Pseudomonas putida*	94	142
Clone_41	WP10	*Pseudomonas putida*	96	142
Clone_42	WP10	*Pseudomonas putida*	95	141
Clone_43	WP10	*Pseudomonas putida*	94	142
Clone_44	WP10	*Pseudomonas putida*	96	142
Clone_45	WP10	*Pseudomonas putida*	91	142

Clone_46	WP14	*Pseudomonas putida*	95	141
Clone_47	WP14	*Pseudomonas putida*	96	142
Clone_48	WP14	*Pseudomonas putida*	94	142
Clone_49	WP14	*Pseudomonas putida*	96	142
Clone_50	WP14	*Pseudomonas putida*	96	143
Clone_51	WP14	*Pseudomonas putida*	95	141
Clone_52	WP14	*Pseudomonas putida*	96	142
Clone_53	WP10	*Pseudomonas putida*	96	142
Clone_54	WP10	*Pseudomonas putida*	96	142
Clone_55	WP10	*Pseudomonas putida*	98	141
Clone_56	WP14	*Pseudomonas putida*	94	141
Clone_57	WP14	*Pseudomonas putida*	96	142
Clone_58	WP14	*Pseudomonas putida*	86	143
Clone_59	WP14	*Pseudomonas putida*	96	143
Clone_60	WP14	*Pseudomonas putida*	95	141
Clone_61	WP14	*Pseudomonas putida*	96	142
Clone_62	WP14	*Pseudomonas putida*	98	141
Clone_63	WP10	*Pseudomonas lundensis*	83	145
Clone_64	WP14	*Pseudomonas gingeri* (*azotoformans*)	96	143
Clone_65	WP10	*Pseudomonas gingeri*	96	143
Clone_66	WP5	*Pseudomonas fluorescens*	85	142
Clone_67	WP5	*Pseudomonas fluorescens*	85	142
Clone_68	WP5	*Pseudomonas fluorescens*	85	142
Clone_69	WP5	*Pseudomonas fluorescens*	90	142
Clone_70	WP5	*Pseudomonas fluorescens*	90	141
Clone_71	WP5	*Pseudomonas fluorescens*	98	182
Clone_72	WP5	*Pseudomonas fluorescens*	85	142
Clone_73	WP5	*Pseudomonas fluorescens*	93	142
Clone_74	WP5	*Pseudomonas fluorescens*	96	143
Clone_75	WP5	*Pseudomonas fluorescens*	91	143
Clone_76	WP5	*Pseudomonas fluorescens*	95	143
Clone_77	WP5	*Pseudomonas fluorescens*	94	142
Clone_78	WP5	*Pseudomonas fluorescens*	96	142
Clone_79	WP5	*Pseudomonas fluorescens*	94	142
Clone_80	WP5	*Pseudomonas fluorescens*	96	142
Clone_81	WP5	*Pseudomonas fluorescens*	95	141
Clone_82	WP5	*Pseudomonas fluorescens*	96	142
Clone_83	WP5	*Pseudomonas fluorescens*	95	141
Clone_84	WP5	*Pseudomonas fluorescens*	94	142
Clone_85	WP5	*Pseudomonas fluorescens*	95	141
Clone_86	WP5	*Pseudomonas fluorescens*	95	141
Clone_87	WP5	*Pseudomonas fluorescens*	94	142
Clone_88	WP5	*Pseudomonas fluorescens*	96	143
Clone_89	WP5	*Pseudomonas fluorescens*	89	142
Clone_90	WP5	*Pseudomonas fluorescens*	83	142
Clone_91	WP5	*Pseudomonas fluorescens*	92	141
Clone_92	WP5	*Pseudomonas fluorescens*	88	141
Clone_93	WP5	*Pseudomonas fluorescens*	91	143
Clone_94	WP10	*Pseudomonas fluorescens*	91	143
Clone_95	WP10	*Pseudomonas fluorescens*	91	143

Clone_96	WP10	*Pseudomonas fluorescens*	91	143
Clone_97	WP10	*Pseudomonas fluorescens*	91	143
Clone_98	WP14	*Pseudomonas fluorescens*	98	141
Clone_99	WP14	*Pseudomonas fluorescens*	91	142
Clone_100	WP14	*Pseudomonas fluorescens*	95	142
Clone_101	WP14	*Pseudomonas fluorescens*	97	142
Clone_102	WP5	*Pseudomonas fluorescens*	94	143
Clone_103	WP14	*Pseudomonas fluorescens*	93	142
Clone_104	WP10	*Pseudomonas fluorescens*	91	143
Clone_105	WP10	*Pseudomonas fluorescens*	94	143
Clone_106	WP10	*Pseudomonas fluorescens*	95	142
Clone_107	WP10	*Pseudomonas fluorescens*	91	143
Clone_108	WP10	*Pseudomonas fluorescens*	91	143
Clone_109	WP10	*Pseudomonas fluorescens*	91	143
Clone_110	WP10	*Pseudomonas fluorescens*	94	143
Clone_111	WP10	*Pseudomonas fluorescens*	95	142
Clone_112	WP10	*Pseudomonas fluorescens*	94	143
Clone_113	WP10	*Pseudomonas fluorescens*	91	143
Clone_114	WP10	*Pseudomonas fluorescens*	91	143
Clone_115	WP10	*Pseudomonas fluorescens*	90	135
Clone_116	WP10	*Pseudomonas fluorescens*	98	142
Clone_117	WP10	*Pseudomonas fluorescens*	91	143
Clone_118	WP10	*Pseudomonas fluorescens*	95	142
Clone_119	WP10	*Pseudomonas fluorescens*	96	143
Clone_120	WP10	*Pseudomonas fluorescens*	94	143
Clone_121	WP10	*Pseudomonas fluorescens*	95	142
Clone_122	WP10	*Pseudomonas fluorescens*	94	143
Clone_123	WP10	*Pseudomonas fluorescens*	94	143
Clone_124	WP10	*Pseudomonas fluorescens*	89	143
Clone_125	WP10	*Pseudomonas fluorescens*	94	143
Clone_126	WP10	*Pseudomonas fluorescens*	94	143
Clone_127	WP10	*Pseudomonas fluorescens*	96	143
Clone_128	WP10	*Pseudomonas fluorescens*	98	142
Clone_129	WP10	*Pseudomonas fluorescens*	94	143
Clone_130	WP10	*Pseudomonas fluorescens*	93	141
Clone_131	WP10	*Pseudomonas fluorescens*	94	143
Clone_132	WP10	*Pseudomonas fluorescens*	98	142
Clone_133	WP10	*Pseudomonas fluorescens*	91	143
Clone_134	WP10	*Pseudomonas fluorescens*	96	143
Clone_135	WP10	*Pseudomonas fluorescens*	91	143
Clone_136	WP10	*Pseudomonas fluorescens*	91	143
Clone_137	WP10	*Pseudomonas fluorescens*	96	143
Clone_138	WP10	*Pseudomonas fluorescens*	93	141
Clone_139	WP10	*Pseudomonas fluorescens*	90	141
Clone_140	WP10	*Pseudomonas fluorescens*	94	142
Clone_141	WP10	*Pseudomonas fluorescens*	95	141
Clone_142	WP10	*Pseudomonas fluorescens*	94	143
Clone_143	WP10	*Pseudomonas fluorescens*	98	147
Clone_144	WP14	*Pseudomonas fluorescens*	94	142
Clone_145	WP14	*Pseudomonas fluorescens*	94	142

Clone_146	WP14	*Pseudomonas fluorescens*	95	142
Clone_147	WP14	*Pseudomonas fluorescens*	95	142
Clone_148	WP14	*Pseudomonas fluorescens*	94	142
Clone_149	WP14	*Pseudomonas fluorescens*	97	142
Clone_150	WP14	*Pseudomonas fluorescens*	91	142
Clone_151	WP5	*Pseudomonas azotoformans*	48	138
Clone_152	WP5	*Pseudomonas azotoformans*	87	143
Clone_153	WP5	*Pseudomonas azotoformans*	94	143
Clone_154	WP5	*Pseudomonas azotoformans*	83	142
Clone_155	WP5	*Pseudomonas azotoformans*	87	143
Clone_156	WP5	*Pseudomonas azotoformans*	87	143
Clone_157	WP5	*Pseudomonas azotoformans*	85	143
Clone_158	WP5	*Pseudomonas azotoformans*	91	143
Clone_159	WP5	*Pseudomonas azotoformans*	84	142
Clone_160	WP5	*Pseudomonas azotoformans*	87	143
Clone_161	WP5	*Pseudomonas azotoformans*	85	143
Clone_162	WP5	*Pseudomonas azotoformans*	95	142
Clone_163	WP5	*Pseudomonas azotoformans*	85	143
Clone_164	WP10	*Pseudomonas azotoformans*	91	142
Clone_165	WP10	*Pseudomonas azotoformans*	64	142
Clone_166	WP10	*Pseudomonas azotoformans*	98	143
Clone_167	WP10	*Pseudomonas azotoformans*	87	143
Clone_168	WP10	*Pseudomonas azotoformans*	94	143
Clone_169	WP10	*Pseudomonas azotoformans*	85	143
Clone_170	WP10	*Pseudomonas azotoformans*	98	143
Clone_171	WP14	*Pseudomonas azotoformans*	87	143
Clone_172	WP14	*Pseudomonas azotoformans*	87	143
Clone_173	WP14	*Pseudomonas azotoformans*	94	143
Clone_174	WP14	*Pseudomonas azotoformans*	88	143
Clone_175	WP10	*Pseudomonas azotoformans*	87	143
Clone_176	WP10	*Pseudomonas azotoformans*	83	142
Clone_177	WP14	*Pseudomonas azotoformans*	91	143
Clone_178	WP14	*Pseudomonas azotoformans*	91	143
Clone_179	WP14	*Pseudomonas azotoformans*	87	143
Clone_180	WP14	*Pseudomonas azotoformans*	91	143
Clone_181	WP10	*Pseudomonas azotoformans*	91	143
Clone_182	WP10	*Pseudomonas azotoformans*	91	143
Clone_183	WP14	*Pseudomonas azotoformans*	89	143
Clone_184	WP14	*Pseudomonas azotoformans*	89	143
Clone_185	WP14	*Pseudomonas azotoformans*	94	141
Clone_186	WP14	*Pseudomonas antarctica*	83	141
Clone_187	WP14	*Pseudomonas antarctica*	83	142
Clone_188	WP14	*Pseudomonas antarctica*	83	142
Clone_189	WP14	*Pseudomonas antarctica*	92	140
Clone_190	WP14	*Sphingobium japonicum*	80	141
Clone_191	WP14	*Sphingomonas* sp	86	142
Clone_192	WP5	*Stenotrophomonas* sp	98	141
Clone_193	WP5	*Stenotrophomonas* sp	95	141
Clone_194	WP5	*Stenotrophomonas* sp	98	141
Clone_195	WP5	*Stenotrophomonas* sp	93	141

Clone_196	WP5	*Stenotrophomonas* sp	94	141
Clone_197	WP5	*Stenotrophomonas* sp	100	143
Clone_198	WP14	*Stenotrophomonas* sp	87	143
Clone_199	WP14	*Stenotrophomonas* sp	98	142
Clone_200	WP14	*Stenotrophomonas* sp	100	142
Clone_201	WP14	*Pseudomonas fluorescens*	85	142
Clone_202	WP14	*Pseudomonas fluorescens*	85	142
Clone_203	WP14	*Pseudomonas fluorescens*	95	141
Clone_204	WP14	*Pseudomonas fluorescens*	93	141
Clone_205	WP14	*Pseudomonas fluorescens*	93	141
Clone_206	WP14	*Pseudomonas fluorescens*	93	141
Clone_207	WP14	*Pseudomonas fluorescens*	95	141
Clone_208	WP14	*Pseudomonas fluorescens*	95	141
Clone_209	WP14	*Pseudomonas fluorescens*	95	142
Clone_210	WP14	*Pseudomonas fluorescens*	94	142
Clone_211	WP14	*Pseudomonas fluorescens*	100	142
Clone_212	WP14	*Pseudomonas fluorescens*	98	142
Clone_213	WP14	*Pseudomonas fluorescens*	96	142
Clone_214	WP14	*Pseudomonas fluorescens*	93	150
Clone_215	WP14	*Pseudomonas fluorescens*	98	142
Clone_216	WP14	*Pseudomonas fluorescens*	98	142
Clone_217	WP14	*Pseudomonas fluorescens*	94	142
Clone_218	WP14	*Pseudomonas fluorescens*	96	142
Clone_219	WP14	*Pseudomonas fluorescens*	96	142
Clone_220	WP5	*errucomicrobiaceae* bacterium CHC1	83	142
Clone_221	WP5	*errucomicrobiaceae* bacterium CHC1	83	141
Clone_222	WP5	*errucomicrobiaceae* bacterium CHC1	85	141
Clone_223	WP5	*errucomicrobiaceae* bacterium CHC1	87	142
Clone_224	WP5	*errucomicrobiaceae* bacterium CHC1	85	141
Clone_225	WP14	*errucomicrobiaceae* bacterium ONA	76	141
Clone_226	WP14	*errucomicrobiaceae* bacterium ONA	87	142
Clone_227	WP14	*errucomicrobiaceae* bacterium ONA	83	141
Clone_228	WP14	*errucomicrobiaceae* bacterium ONA	82	128
